# RAR-related orphan receptor alpha and the staggerer mice: a fine molecular story

**DOI:** 10.3389/fendo.2023.1300729

**Published:** 2024-05-03

**Authors:** Aradhana Rani

**Affiliations:** ^1^ Medical Biochemistry, Jawaharlal Institute of Postgraduate Medical Education and Research (JIPMER), Pondicherry, India; ^2^ Human Resource Development and Management, Indian Institute of Technology (IIT) Kharagpur, West Bengal, India; ^3^ Immunology, King’s College London, London, United Kingdom

**Keywords:** *RORα*, staggerer mice, melatonin, circadian rhythm, embryogenesis, nutrition

## Abstract

The retinoic acid-related orphan receptor alpha (RORα) protein first came into the limelight due to a set of staggerer mice, discovered at the Jackson Laboratories in the United States of America by Sidman, Lane, and Dickie (1962) and genetically deciphered by Hamilton et al. in 1996. These staggerer mice exhibited cerebellar defects, an ataxic gait, a stagger along with several other developmental abnormalities, compensatory mechanisms, and, most importantly, a deletion of 160 kilobases (kb), encompassing the *RORα* ligand binding domain (LBD). The discovery of the staggerer mice and the subsequent discovery of a loss of the LBD within the *RORα* gene of these mice at the genetic level clearly indicated that RORα’s LBD played a crucial role in patterning during embryogenesis. Moreover, a chance study by Roffler-Tarlov and Sidman (1978) noted reduced concentrations of glutamic acid levels in the staggerer mice, indicating a possible role for the essence of a nutritionally balanced diet. The sequential organisation of the building blocks of intact genes, requires the nucleotide bases of deoxyribonucleic acid (DNA): purines and pyrimidines, both of which are synthesized, upon a constant supply of glutamine, an amino acid fortified in a balanced diet and a byproduct of the carbohydrate and lipid metabolic pathways. A nutritionally balanced diet, along with a metabolic “enzymatic machinery” devoid of mutations/aberrations, was essential in the uninterrupted transcription of RORα during embryogenesis. In addition to the above, following translation, a ligand-responsive RORα acts as a “molecular circadian regulator” during embryogenesis and not only is expressed selectively and differentially, but also promotes differential activity depending on the anatomical and pathological site of its expression. RORα is highly expressed in the central nervous system (CNS) and the endocrine organs. Additionally, *RORα* and the *clock* genes are core components of the circadian rhythmicity, with the expression of *RORα* fluctuating in a night–day–night sigmoidal pattern and undoubtedly serves as an endocrine-like, albeit “molecular–circadian regulator”. Melatonin, a circadian hormone, along with tri-iodothyronine and some steroid hormones are known to regulate RORα-mediated molecular activity, with each of these hormones themselves being regulated rhythmically by the hypothalamic–pituitary axis (HPA). The HPA regulates the circadian rhythm and cyclical release of hormones, in a self-regulatory feedback loop. Irregular sleep–wake patterns affect circadian rhythmicity and the ability of the immune system to withstand infections. The staggerer mice with their thinner bones, an altered skeletal musculature, an aberrant metabolic profile, the ataxic gait and an underdeveloped cerebellar cortex; exhibited compensatory mechanisms, that not only allowed the survival of the staggerer mice, but also enhanced protection from microbial invasions and resistance to high-fat-diet induced obesity. This review has been compiled in its present form, more than 14 years later after a chromatin immunoprecipitation (ChIP) cloning and sequencing methodology helped me identify signal transducer and activator of transcription 5 (STAT5) target sequences, one of which was mapped to the first intron of the *RORα* gene. The 599-base-long sequence containing one consensus TTCNNNGAA (TTCN_3_GAA) gamma-activated sequence (GAS) and five other non-consensus TTN_5_AA sequences had been identified from the clones isolated from the STAT5 target sites (fragments) in human phytohemagglutinin-activated CD8+ T lymphocytes, during my doctoral studies between 2006 and 2009. Most importantly, preliminary studies noted a unique *RORα* expression profile, during a time-course study on the ribonucleic acid (RNA), extracted from human phytohemagglutinin (PHA) activated CD8+ T lymphocytes stimulated with interleukin-2 (IL-2). This review mainly focuses on the “staggerer mice” with one of its first roles materialising during embryogenesis, a molecular-endocrine mediated circadian-like regulatory process.

## Introduction

**Figure 1:**
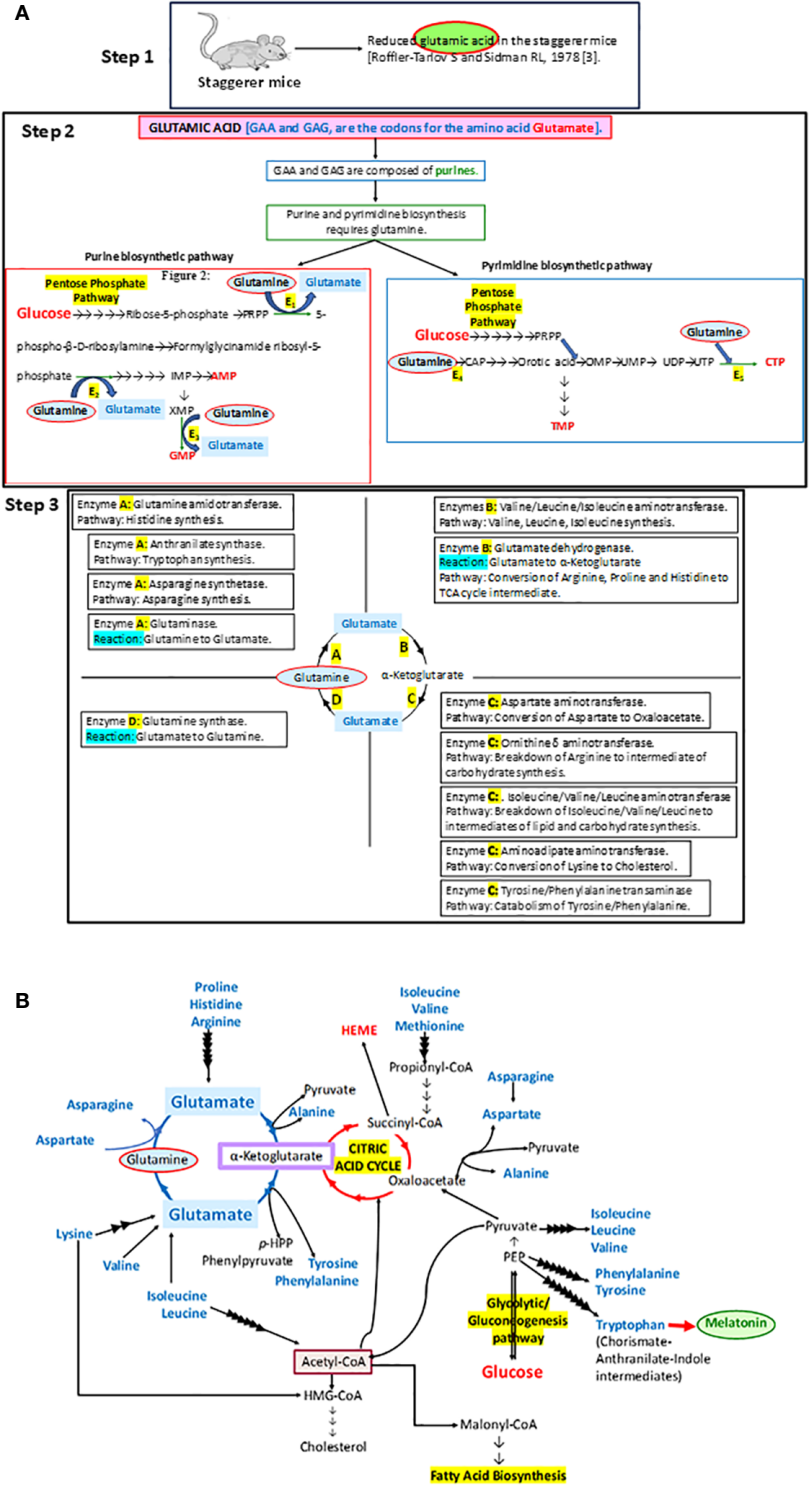
**(A)** A step-by-step analysis of the observations by Roffler-Tarlov and Sidman (1978) (3). The enzymes E1, E2, E3, E4, and E5 in step 2 that require glutamine as a substrate in the synthesis of purines and pyrimidines stand for phosphoribosyl pyrophosphate (PRPP) glutamyl amidotransferase (E1), formylglycinamide ribosyl-5-phosphate synthetase (E2), xanthosine monophosphate (XMP) transamidinase (E3), cabamoyl phosphate (CAP) synthase II (E4), and cytosine triphosphate (CTP) synthase (E5), respectively. PRPP is phosphoribosyl pyrophosphate, IMP is inosine monophosphate, GMP is guanosine monophosphate, AMP is adenosine monophosphate, CAP is carbamoyl phosphate, OMP is orotidine monophosphate, UMP is uracil monophosphate, UDP is uracil diphosphate, UTP is uracil triphosphate, CTP is cytosine triphosphate, and TMP is thymine monophosphate. Some of the other enzymes involved in metabolic pathways, as part of the glutamine→glutamate→α ketoglutarate cycle: A, B, C and D, are denoted in the four quadrants at step 3. **(B)** The importance of the glutamine→glutamate→α ketoglutarate cycle and its association with carbohydrate, lipid, and protein metabolism has been illustrated in this diagram.

It was a set of staggerer mice that drew the attention of Sidman et al. in 1962 to their underdeveloped cerebellar cortex (1). The staggerer mice were a set of mouse mutants, maintained at the Jackson Laboratory, after an F1 female (BALB/cHm crossed with C3H/HeJ) had bred with the male of an obese (Lep^ob^) stock mouse (https://www.jax.org/strain/000237). Sixteen years later, in 1978, Landis and Sidman studied the developmental stages of the staggerer mice mutants and noted pathological changes in the cerebellar cortex of these mice (2). Earlier in 1978, Roffler-Tarlov and Sidman noted reduced concentrations of glutamic acid in the staggerer mice (3). The glutamine→glutamate→α-ketoglutarate cycle stands at the crossroads of carbohydrate, protein, and lipid metabolism, serving as an essential precursor to the synthesis of purines and pyrimidines, which is the mainstay in the uninterrupted synthesis of genes on chromosomes (**Figure 1**). It was no wonder then that Roffler-Tarlov and Sidman noted reduced glutamic acid, encoded by the purine codons GAA and GAG, which might have affected the transcription and translation of the ligand binding domain (LBD) of *RORα* (3, 4). However, it was not until 1996 when Hamilton et al. deciphered the genetic reasoning behind the characteristic clinical observations in the staggerer mice—the deletion of 160 kilobases on chromosome 9—that resulted in a truncated version of the RORα protein, devoid of its LBD (4). The deletional mutation affecting the LBD of the RORα gene led to the birth of the staggerer (sg^−^/sg^−^) mice exhibiting symptoms associated with a loss of function to the downstream cascade of signaling events promoted by ligand-induced RORα. Essentially a deletion of RORα’s LBD region manifested itself as pronounced developmental and metabolic alterations in the cerebellum, adipose tissue, bones, cardiac and skeletal muscles, liver, thymus, and spleen of the staggerer mice (1, 5–9).

The staggerer mice, with a defunct RORα LBD that led to the disruption to RORα-mediated crucial genetic networks, were endowed with specific redundant genetic mechanisms that permitted viability of these mice. The redundancy of RORα to the transcription factors DHR3, RARα1, RXRα, Rev-ERBα, T3Rβ, PPARα, VDR, or GR, at specific anatomical and pathological sites, upon activation with a specific ligand, was essential to the survival and longevity of the staggerer mice (10–12). The activation of RORα and their redundant transcription factors within specific cells and tissues, with the specific ligand, leads to the activation of a cascade of signaling molecules and the organization of cells within tissues during the stages of embryogenesis, an absence or the inability of which leads to the staggerer mice (4, 10–14). The known ligands of RORα are melatonin, tri-iodothyronine (T3), the steroid hormones (estrogen, testosterone and the adrenal gland hormones, cortisol, and adrenocorticotrophic hormones), cholesterol and cholesterol derivatives (15–20). These ligands, sometimes known as hormones, are mainly under the control of the hypothalamic–pituitary axis (HPA) and function as an integral part of the circadian rhythm. In response to the cyclical release of hormones by the HPA, a “molecular circadian clock” operates within cells, composed of the *clock* genes and *RORα* (21–26). Sato et al. (2004) along with Akashi et al. (2005) clearly substantiated *RORα* as a circadian clock component (21, 23).

In essence, the loss of RORα, a crucial molecular circadian clock gene, led to a tsunamic effect on RORα-dependent molecular events and functions. Sidman et al. (1962) more precisely described the staggerer mice as recognized with a “staggering gait, mild tremor, hypotonia, and small size”, and that their “cerebellar cortex is grossly underdeveloped, with too few granule cells and unaligned Purkinje cells” (1). Moreover, glutamic acid—an amino acid intermediate of the essential and non-essential amino acids (aspartate, alanine, isoleucine, leucine, valine, methionine, asparagine, phenylalanine, tyrosine, arginine, lysine, histidine, and proline), carbohydrate metabolism at the α-ketoglutarate and oxaloacetate intermediate stages (Krebs tricarboxylic citric acid cycle pathway), and lipid metabolism (free fatty acids metabolize into acetyl-CoA→citrate→α-ketoglutarate→glutamate)—was noted to be reduced in the staggerer mice (**Figure 1**) (3). Thus, nutrition and the ability of an enzymatic machinery in the pre- and post-natal cells to assimilate and metabolize carbohydrates, lipids, proteins, and nucleotides play an important role in the development of wild-type mice without any mutations. An unhealthy diet, bereft of the essential amino acids, is the main cause of a disrupted molecular–circadian rhythm, dysregulated endocrine HPA, anomalous metabolism, mutations, and an immune system susceptible to infections.

This review had been conceptualized due to *RORα* being identified as a signal transducer and activator of transcription 5 (STAT5) target gene during my doctoral studies at King’s College London, although the review in its present form has been compiled after literature surveys led me to the staggerer mice and melatonin as the keywords, during searches regarding the genetics of *RORα*.

## RORα: gene and protein structure

**Figure 2:**
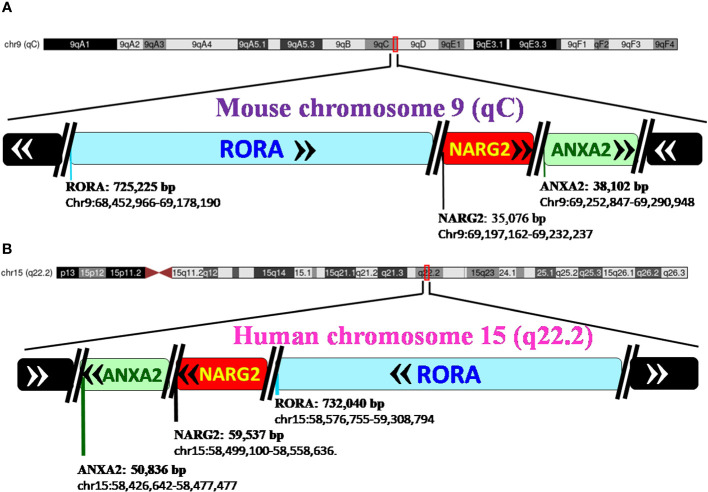
Pictorial representation of the chromosomal mapping of RORα gene in mice and humans. **(A)** RORα in the mouse maps to qC on chromosome 9, and its length and precise location are denoted below the name of the gene, as per the UCSC Genome Browser on Mouse Feb 2006 (NCBI36/mm8). The gene transcribes away from the centromere. RORα is followed by NARG2 and ANXA2, all the three genes being transcribed in a direction away from the centromere. **(B)** In humans, RORα maps to q22.2 on chromosome 15, as per the UCSC Genome Browser on Human Mar. 2006 (NCBI36/hg18) build, and transcribes towards the centromere (28). [The length of the gene is mentioned alongside the name of the genes as in panel **(B)** RORA: 732,040 base pairs and the region that the gene spans on the chromosome, as chr15:58,576,755-59,308,794. In humans, the three genes, RORα, NARG2, and ANXA2, transcribe themselves in a 5′–3′ direction towards the centromere. RORA, NARG2, and ANXA2 refer to the isoform 1 of each of these genes.].

RORα belongs to a group of nuclear receptor (NR) superfamily of transcription factors that elicit their response upon binding to a hormone response element (HRE), more commonly known as a RORE site. The basic structure of RORα, like all other members of the nuclear family of receptors, contains the following domains: an N-terminal transactivation domain (NTTD), a DNA binding domain (DBD) that determines its specificity for specific DNA sequences within genes, a hinge domain, and a carboxy terminal LBD that dictates crucial conformational changes in the structure (27). A grouping of the NR family of proteins, based on the HUGO nomenclature, is listed in **Supplementary Table 1** (HUGO Gene Nomenclature Committee). *RORα* is located on the long arm, at the q22.2 loci on human chromosome 15, as per the human assembly, March 2006 build NCBI36/hg18 (www.ncbi.nlm.nih.gov 28). The carboxy terminus on the RORα protein and gene, in mice and humans, is close to two other genes of significance: NMDA receptor-regulated 2 (*NARG2)* and Annexin A2 (*ANXA2)* (4, 28). A schematic representation of the chromosomal mapping of *RORα* in humans and mice is depicted in **Figure 2**. Notably, while *RORα* is transcribed in a 5′ to 3′ direction away from the centromere in the “nocturnal mouse”; in the “diurnal humans”, *RORα* is transcribed in a direction towards the centromere (28). There are at least four known isoforms of *RORα*—*RORα1, RORα2, RORα3*, and *RORα4*—that belong to the group 2 NRs and most studies have noted a predominant role for *RORα1* and *RORα4* (27–31). The four isoforms of *RORα* as per the UCSC genome browser databases are listed in **Table 1** (28). Each of these four isoforms display differential activities depending on their differential expression in specific cells within specific tissues (14, 15, 21–24).

**Table 1:** The four isoforms of RORα, and their respective loci on human chromosome 15 (28).

March 2006, NCBI36/hg18	Transcript/Coding region	Location on Chromosome 15	Size (base pairs)	Exon count
RORα1	Transcript (including UTRs)	chr15:58,576,755-59,308,794	732,040	11
	Coding region	chr15:58,576,946-59,308,709	731,764	11
RORα2	Transcript (including UTRs)	chr15:58,576,755-58,707,024	130,270	12
	Coding region	chr15:58,576,946-58,706,865	129,920	12
RORα3	Transcript (including UTRs)	chr15:58,576,755-58,707,024	130,270	11
	Coding region	chr15:58,576,946-58,706,865	129,920	11
RORα4	Transcript (including UTRs)	hg18 chr15:58,576,755-58,671,999	95,245	10
	Coding region	chr15:58,576,946-58,671,908	94,963	10

*RORα* was first cloned and functionally characterized by Giguere et al. in 1994 and they identified a notable difference, in that the N-terminal domain dictates the specificity for DNA binding by each of the variants (11). X-ray crystallography at a resolution of 1.63 Å and 2.2 Å identified cholesterol and its derivative as RORα’s natural ligands at the LBD of RORα (19, 20). A study by McBroom et al. (1994) revealed that the hinge region, located between the DBD and the LBD, determines the angle of bend that occurs to stabilize RORα–DNA interactions (29). Of essence is also the study where sequence alignment studies detected homology in the DBD, between RORα and several other NRs: Retinoid X receptor (RXRα), V-ErbA-Related Protein 1 (Rev-Erbα), thyroid hormone receptor (T3Rβ), peroxisome proliferator-activated receptor (PPARα), vitamin D receptor (VDR), and the glucocorticoid receptor (GR) (11). One of the reasons behind the ability of staggerer mice to survive embryogenesis past adulthood is a result of the compensatory mechanisms exercised through the redundant role sharing between RORα and few other NR family members. It was the DNA binding sequence of *Rev-Erbα* that is very similar to *RORα1* and *RORα2* (11, 12). Sequence alignment of *RORα1* with *Rev-Erbα* showed 60% similarity of its DBD to RORα1 and only 30% of its LBD to RORα1 (11, 12). Again, amino acid sequence alignment revealed similarities between the mouse *Rev-Erbβ*, rat *Rev-Erbα*, human *RORα*, and rat *RZRβ* (10). *Rev-Erbβ*, *Rev-Erbα*, and *RORα1* bind to the same extended binding site (10). Additionally, Giguere et al. go on to state that the RORα and Rev-ErbAα-binding sites are practically indistinguishable, and the two receptor systems should be expected to control overlapping gene networks (11). In support of the above, a comprehensive study by Mukherjee et al. (2021) has clearly demonstrated that RORα along with the other NRs, Rev-Erbα, PPARβ, Erβ, and PPARα are expressed uniquely in the intestinal compartments, ileum, and colon, although there is a certain degree of redundancy between these NRs (12).

Basically, RORα mediates its actions through the activation of the ligand–RORα complex, which then binds to consensus sequences within target genes, namely, the ROR response elements (ROREs) (11). The ROREs are composed of a 6-bp-long A/T-rich sequence followed by (A/T)GGTCA (11). It is important to note that Odawara et al. identified a novel RORα binding site within the aromatase gene, more specifically at exon 1.4 in MCF7 cells, although occupancy by RORα within the exon would possibly be expected to block the transcriptional activity of the aromatase gene (30). Upon ligand-induced RORα binding to the RORE sites within genes, transcriptional activity ensues, which is attained through post-translational and structural modifications, aided through the binding of an array of proteins to the regulatory sites of genes.

### RORα: post-translational modifications

**Figure 3:**
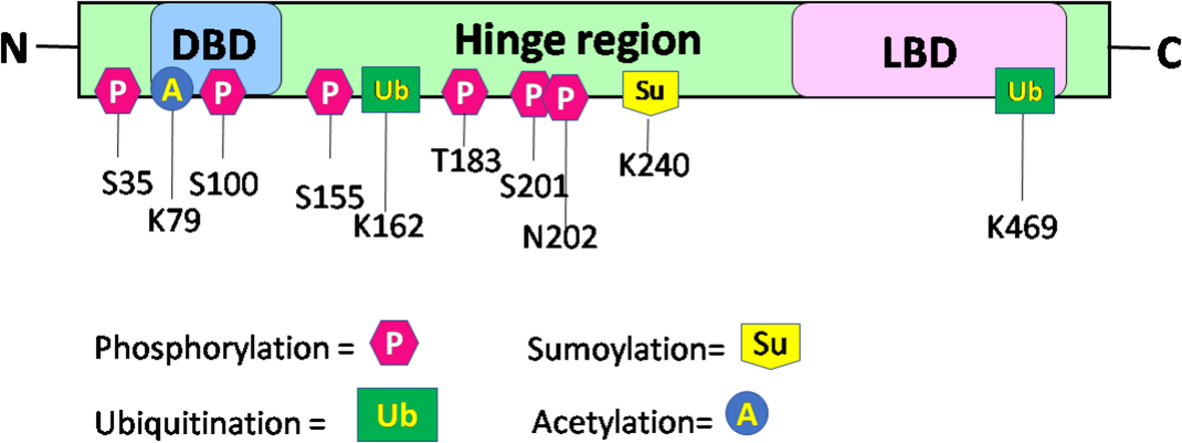
Schematic structure and post-translational modification sites of RORα in humans. This figure for RORα has been modified and reproduced from www.phosphosite.org.

Upon binding of a ligand to the LBD of the RORα gene, a conformational change ensues, thereby promoting its binding to regulatory regions within their target genes and subsequent effects on the regulation of embryogenesis, with modulation of a molecular–circadian rhythm being one of the first and foremost in the cascade of regulatory processes affected (21, 23). RORα, akin to other transcriptional factors, promotes its action through post-translational modifications: phosphorylation, acetylation, ubiquitination, and SUMOylation (27). The post-translationally modified sites of RORα are depicted in **Figure 3**. According to Hornbeck et al. (2015), RORα is phosphorylated at S35, S100, S155, T183, S201, and N202; ubiquitinated at K162 and K469; acetylated at K79; and SUMOylated at K240 (27).

Post-translational modification of RORα promotes the activation or repression of its intrinsic activity and the target genes, thereby promoting successful embryogenesis, growth, and development (1, 2, 4, 31–35). Phosphorylation of RORα at serine 35 is promoted by the activation of protein kinase C (PKC) (31). Ermisch et al. identified protein kinase A (PKA) as one of the kinases phosphorylating RORα and a mutation at the phosphorylating site led to an abolishment of the phosphorylating capacity by PKA at that site (32). Ermisch et al. subsequently concluded that RORα4 is regulated by PKA and calcium/calmodulin-dependent protein kinase IV (CaMK-IV) (32). Extracellular signal-regulated kinase 2 (ERK-2) is another RORα4 phosphorylating protein, where the hinge region of RORα4 contains an ERK-2 binding motif at the threonine residue 128, and a mutation of T128A leads to enhanced transcriptional activity (33). Using the HEK293 cells, RORα was identified to be phosphorylated by protein kinase C alpha (PKCα), within the N terminal domain (31). RORα is also phosphorylated at S100 (34). More specifically, chromatin immunoprecipitation (ChIP) studies, Western blotting, and luciferase assays performed on the S100 site of RORα, using transfection of the hRORαWT100, hRORαS100A (alanine = A), or hRORαS100D (aspartate = D) sequences into pGL3 vectors containing the SULT1e1 promoter, validated the phosphorylation of RORα at the S100 locus (34). While RORα is phosphorylated by ERK-2, PKA, and PKCα, it is also SUMOylated by small ubiquitin-like modifier 1 (SUMO1) and SUMO2 at the Lysine 240 residue, by the protein inhibitor of activated STAT (PIAS) proteins (**Figure 3**) (27, 31–35). Furthermore, RORα is acetylated at K79 and ubiquitinated at K162 and K469, respectively (27). In addition to these modifications within the RORα protein, which generates a structural configuration aimed at accomplishing distinct functions within distinct cells in a tissue, RORα also promotes the recruitment of transcriptional coregulators.

### RORα: transcriptional coregulators and regulation

RORα mediates its actions through its association with co-activators and co-repressors that act as a conglomeration of proteins to promote transcriptional functions, with one of its first roles materializing during embryogenesis. The activation/repression of upstream/downstream genes is determined through a fine cross-talk between transcriptional coregulators that regulate *RORα*. RORα is regulated by glucocorticoid receptor-interacting protein-1 (GRIP-1), with GRIP1 itself being phosphorylated by glucocorticoid-mediated glucocorticoid receptor (GR) interactions (36, 37). Atkins et al. (1999), using a two-hybrid screen, had identified *RORα* coactivators that bind as a complex of proteins: GRIP-1 and peroxisome proliferator-activated receptor (PPAR)-binding protein (PBP/TRAP220/DRIP205) (36). The same study also identified TRIP-1, TIF-A, TRIP230/TRIP-11, and PBP as few other RORα modulators (36). Subsequently, Ji Min Lee et al. in 2001 identified β-catenin and GRIP1, along with several other protein binding partners of RORα, with molecular weights of 270, 220, 160, 140, 65, and 45 kDa, respectively (31). A *GRIP1* knockout study defined the importance of GRIP1 during embryogenesis (38). Another co-activator of *RORα* is PPARG coactivator 1 alpha (PGC1α), a regulator of energy metabolism (39). The PGC1α-RORα pathway regulates the circadian genes BMAL1, clock, and Rev-ERBα, possibly through a self-regulatory “molecular circadian rhythm” (39, 40). While the coregulator PGC1α activates the transcription of *RORα*, Hairless (Hr) acts as a repressor of *RORα*’s transcriptional activity (40, 41). In a separate study, p300 and PGC1α were identified as the upstream coactivators of RORα; these coactivators along with RORα regulate caveolin-3 (CAV3) and the enzyme carnitine palmitoyltransferase-1 (CPT1) in the muscle (42). While some transcription factors act as coregulators of *RORα*, RORα itself serves as a coregulator.

In the liver, RORα acts as a coactivator with SRC2 to regulate the functions of the enzyme Glucose-6-phosphatase (43). RORα also binds to the promoters *of Slc1a6, Itpr1, Pcp4*, and *Pcp2* to regulate their expression (44). The string database has assembled and organized a few of the proteins that interact with RORα, some of them as of July 2023 are as follows: NPAS2, CLOCK, NRIP1, ARNTL, HIF1A, KAT5, WNT5A, STAT3, BCL6, and BATF (www.string-db.org). The protein N-MYC expressed highly in the brain is regulated by RORα (45, 46). The staggerer mice, an example of a molecular–endocrine–circadian rhythm dysregulated model of mice, expressed reduced levels of the sonic hedgehog (Shh) transcript and RORα was ascertained to bind to a RORE sequence in the promoter of Shh, along with the coactivators β catenin and p300 (44). Studies in mice have shown that RORα, ataxin 1 (ATXN1), and the RORα coactivator lysine acetyltransferase (Tip60) exist as a complex of proteins, whereby cerebellar development is affected (47–49). Specific isoforms of RORα, in the presence/absence of specific activators/repressors, undergo specific modifications and perform specific functions, in the specific tissue. The RORα1 isoform interacts with co-repressors nuclear receptor corepressor 1 (NCOR1) and nuclear receptor corepressor 2 (SMRT/NCOR2) (48). It is again the RORα1 isoform that forms a complex with myogenin (myoD) and p300 (50). While the network of proteins regulating *RORα* and its isoforms are dependent on the pathological site of action, the sequence of genes and transcription factors regulated by RORα and its isoforms are also dependent on the site of action.

### RORα: gene expression profiling studies

*RORα* is expressed differentially depending on the activating ligand, the anatomical site of action, the time of the day, and the stage of embryonic development. The staggerer mice with a deleted LBD within the RORα had affected the cerebellar cortex of the mice (1, 4, 49). In agreement with the findings of Sidman et al. (1962) and Hamilton et al. (1996), along with recent genomic studies on the human brain, RORα is expressed copiously in the cerebellum of humans (1, 4, 46). The normalized transcript expression (nTPM) data for *RORα* in the brain, available from the Human Protein Atlas, revealed the highest expression in the cerebellum (nTPM = 51.1), followed by the thalamus (49.6), midbrain (36), amygdala (19.9), cerebral cortex (13.1), hypothalamus (12.6), and basal ganglia (11.7), respectively. Few other organs presenting with high expression of RORα were the adrenal gland (nTPM = 38), liver (28.7), bone marrow (17.5), skeletal muscle (14.4), and the thyroid gland (12.8) (Human Protein Atlas dataset) (46). Northern blot analysis done on several human tissues indicated that *Rev-Erbβ, Rev-Erbα*, and *RORα1* are expressed in the brain and skeletal muscle, while *Rev-Erbβ* and *Rev-Erbα* are expressed in the lung, liver, and kidneys too (10). Gene expression analysis on skeletal muscle, liver, and epididymal fat of staggerer mice reported diminished expression of *RORα1* and *RORα4*, when compared to the WT mice (7). Understandably, the four isoforms of RORα perform independent functions and, under altered circumstances, play redundant roles. Thus, although *RORα* is expressed in several other organs, the mountain of evidence for the role of RORα, an endocrine-like-mediator of a "molecular circadian rhythm", in organogenesis is irrefutable (1, 49).

Activation of RORα upon the formation of a ligand–RORα complex leads to the binding of RORα to RORE sequences, thereby mediating the activation/repression of several genes. Some of the genes known to be activated/repressed are: *NMYC, CAV-3, CABP3, FAS, ADRP, ACS4, SCD1, SCD2, VEGF, HIF1α, ALOX5, apoa5, srebp1, ampk, apocIII, apocI, shh, slc1a6, Itpr1, pcp4, pcp2, CIDEC, CIDEA, GPAM/GPAT1, AGPAT9, MOGAT1, ACOT3, acyl-coenzyme A thioesterase 4 (ACOT4), FABP5, ADFP/PERILIPIN 2, LPIN2, ANGPT14, FGF21, CYP19A1, MIF, ABCA1, SEMA3E, NEPH, ADCY8, NR2F1, Netrin G1, CD47, CSPG5, LXRα, CYP7B1, IkBα, c-jun, E4BP4, IL-6, REG3Y, STAT3*, and *TNFα* (42, 44, 45, 51–61). A list of the genes regulated by RORα is listed in **Supplementary Table 2**.

## RORα: circadian rhythm–HPA

A molecular circadian rhythm that regulates the expression of *RORα* through the developmental stages of life is a determinant of uninterrupted embryogenesis, the birth of a fetus, without cerebellar defects, and the obvious stagger (1, 2). Years later, the research by Akashi and Takumi (2005) and Sato et al. (2004) then laid the foundation for a role of RORα as a core component of the mammalian circadian clock (21, 23). In 2006, Yang et al. cemented the findings of Akashi and Takumi (2005) and Sato (2004) when they studied the rhythmic expression of *RORα, RORγ, Clock, Bmal1, Per2, Cry1, Rev-ERBα, Rev-ERBβ, PPARγ, PPARα, PPARδ, PGC1α*, and *TRα* in the white adipose tissue (WAT), brown adipose tissue (BAT), liver, and skeletal muscle of mice during a 28-h day–night period (21, 23, 40). While the expression of RORα was the highest in the WAT, at the 12-h Zeitbeger time (ZT), the well-known circadian genes, *Clock* and *Bmal1*, dipped to their lowest (40). Earlier, *Clock, Bmal1, Per1*, and *Per2* had been identified as circadian rhythm clock genes (62).

**Figure 4:**
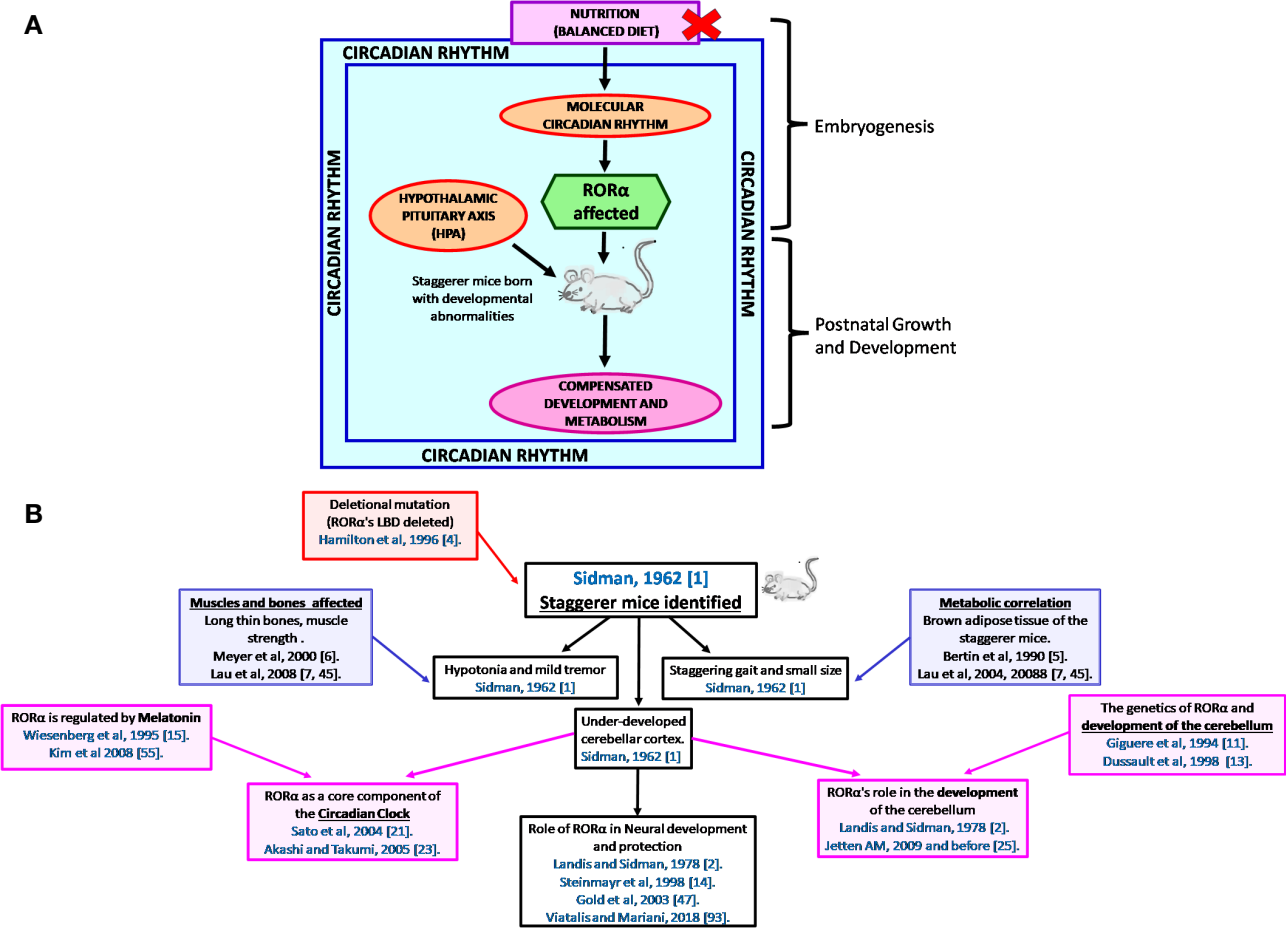
**(A)** A schematic representation of the thought process behind the birth of the staggerer mice and its compensatory mechanism. A nutritionally challenged/imbalanced diet led to error-prone DNA replication, unable to direct the systematic regulation of the molecular circadian events, with the RORα gene being affected. A mutated RORα ligand binding domain led to the birth of the staggerer mice. The staggerer mice were endowed with a set of compensatory mechanisms to withstand the mutation, through adiposity resistant metabolic pathways, along with following alternate transcriptional (Rev-ERBα/Rev-ERBβ) pathways. **(B)** A flowchart of the milestones after the discovery of the staggerer mice in 1962 by Sidman, Lane, and Dickie in 1962 (1). The foundations of this review lie on the substantial research findings by all the authors mentioned in this flowchart (1, 2, 4–7, 11, 13–15, 21, 23, 25, 45, 47, 55, 70).

It is the rhythmic circadian cycle that plays a crucial role in maintaining balance and normal gait, which was absent from the staggerer mice. In 2017, three researchers, Jeffrey Hall, Michael Rosbash, and Michael Young, were awarded the Nobel Prize for their studies on the circadian gene “period” (63, 64). The original concept of circadian rhythm was said to have been derived from the rotation of the earth around its axis in 24 h. The circadian rhythm is responsible for several rhythmic variations observed in humans, with the hormonal and temperature fluctuations, the metabolic changes, and the sleep–wake hours being some of the obvious rhythmic changes observed between various times of the day (21, 22, 62, 65, 66). Some of the external factors that maintain the biological rhythm are light, diet, sleep and wake hours (time of the day), and sedentary/active lifestyle, among many other known and unknown factors (65–68). A synchronized HPA, with positive and negative feedback mechanisms, allows for specific hormonal release at specific times of the day, thereby regulating a spontaneous and balanced circadian rhythm, and *vice versa* (66, 67). The suprachiasmatic nucleus (SCN) of the hypothalamus serves as a pacemaker of circadian oscillation (66, 67). RORα is expressed in the SCN of the hypothalamus and the transcript levels of RORα follow a cyclical day–night harmonic trend (40, 66). After numerous studies on RORα and the staggerer mice, Vitalis and Mariani in 2018 had concluded that “RORα acts initially in various aspects of brain construction and later on in neuroprotection” (69). My hypothesis, based on already published data, that led to the birth of the staggerer mice is depicted pictorially in **Figures 4A, B**. Some of the hormones known to affect RORα regulation rhythmically/arrhythmically are discussed below.

### RORα: melatonin

The circadian rhythm is mainly maintained through an HPA-regulated homeostatic mechanism, composed of hormones, one of which is melatonin, released by the pineal gland. Although melatonin is predominantly secreted by the pineal gland, melatonin is also produced by the lymphocytes and the bone marrow (71, 72). Melatonin is a metabolite of the nutritionally essential amino acid tryptophan. The essential amino acid tryptophan is hydroxylated to 5-hydroxytryptophan, which is further metabolized to serotonin and melatonin. Melatonin was identified as a ligand for RORα1, when studying the effects of the synthetic compound CGP52608 (15). The circadian hormone melatonin, also known as the “hormone of darkness”, was identified as a transcriptional regulator of RORα and RORα1 (15, 16, 54). Eun-Jin Kim et al. (2008) successfully demonstrated that melatonin enhances the transcriptional activity of RORα1, RORα4, HIF1α, and VEGF (54). Melatonin is a circadian rhythm hormone, fluctuating rhythmically, with a peak occurring during the early hours after midnight, and serves as a neuroprotective hormone through the brain glutathione peroxidase (73–75). The antioxidant enzymes glutathione peroxidase 1 (gpx1) and peroxiredoxin 6 (prx6) promote neuroprotection through the human RORα1 (76). In MCF7 cells, melatonin acts on MT1 and RORα to inhibit cell proliferation (77, 78).

### RORα: steroidogenic hormones

An interesting study by Komatsubara et al. (2017) deciphered several interactions between adrenocortical steroid hormones, melatonin, BMP-4, and catecholamine synthesis in rat pheochromocytoma (PC12) cells (79). Cholesterol is a precursor to the steroidogenic hormones and Kallen et al. (2002) discovered cholesterol as a natural ligand of RORα (19, 80). RORα is postulated to be constitutively activated in a specific conformation, and evidence from HepG2 cells treated with cholesterol sulfate, 22®-hydroxycholesterol, and 7-dehydrocholesterol demonstrated enhanced expression of RORα4, HIF-1α, and VEGF (19, 54). Assays to study the effects on RORα’s LBD identified maximum activation of RORα by cholesterol sulfate, followed by 7-dehydrocholesterol, cholesterol, epicholesterol, and cholestenol, respectively (19). An interesting study by Kallen et al. (2004) identified cholesterol 3-o-sulfate as a stronger ligand of RORα, when compared to cholesterol, the reason being additional hydrogen bonds at the RORα LBD site (20). The angle of bend between the DBD and the LBD that is plausible due to the hinge region and leads to stabilization of RORα complexes is determined by specific ligands and hormones that act either transiently or constitutively (29). Since cholesterol is a precursor of the steroid hormones that serve as critical mediators in the HPA axis and is a natural ligand of RORα, any synthetic modifications to cholesterol might have deleterious effects on the pathophysiology of the RORα regulatory pathway (80). However, estradiol (E2), a natural cholesterol metabolite, was established to activate RORα and plays a crucial role in bone mineralization (18). Osteopenic bones were a hallmark of the staggerer mice (6). E2, the female reproductive hormone, activates RORα, and the estrogen receptor β (ESR2) along with estrogen receptor-related protein 3 (ESRRG) were identified as potential RORα target genes by Sarachana and Wu (2013) (18, 81). The same study also identified progesterone receptor membrane component 2 (*PGRMC2*), the hydroxysteroid 17-beta dehydrogenase 3 (*HSD17B3*), and hydroxysteroid 17-beta dehydrogenase 10 (*HSD17B10*) as RORα targets in their genome-wide study on a neuronal cell line (81). Moreover, upon stimulation of MC3T3-E1 osteoblastic cells with E2, there was a RORα-mediated upregulation of bone morphogenetic protein 2 (BMP2) and RUNX family transcription factor 1 (RUNX1) (18). Testosterone, another cholesterol metabolite, serves as a ligand for RORα in the skeletal muscles (82).

The staggerer mice showed enhanced responsiveness to novelty stress when compared to the wild-type mice, with enhanced levels of adrenocorticotropic hormone (ACTH) (83). Basically, the hormones produced by the adrenal cortex are controlled by the corticotropin-releasing hormone (CRH) and the anterior pituitary, under positive and negative feedback mechanisms. At an nTPM of 38, the adrenal gland expresses RORα only next, after the cerebellum (51.1) and the thalamus (49.6) (46).

Apart from the HPA-regulated positive and negative feedback mechanisms, cyclical variations influenced by seasonal changes, varied stages of development/aging, sleep, and psychological changes are among few of the known factors that affect RORα expression and regulatory pathways. Under stressed conditions, the staggerer mice exhibited enhanced endocrine activity, in a study of the ACTH levels in these mice (83). Additionally, the expression of cytochrome P450 family 7 b1 (*CYP7B1*), which is a RORα target gene, was reduced in the staggerer mice (59, 81). The cytochrome p450 family of enzymes are present in the adrenals as well as the liver and a number of genes in this family of proteins were identified as putative RORα target genes (81). The cytochrome family of genes identified as part of the Sarachana and Wu study were as follows: *CYP2R1, CYP7B1, CYP2D7P1, CYP26B1, CYP26A1, CYP27C1, CYP19A1, CYP11A1, CYP2B6, CYP2B6, CYP2A13, CYP2U1, CYP2A7, CYP4A22*, and *CYP4F3* respectively (81).

### RORα: eicosanoid hormones

Since the RORα–HPA molecular circuitry is a complex association, some of the other pathways regulated are the NF-κB-IkBα and TNFα-COX2 (60, 84). RORα1 activates the NF-kB-IkBα pathway, upon binding to a IkBα RORE, although melatonin-induced RORα inhibits NF-kB activity (60, 84). RORα1, through a TNFα-mediated pathway, leads to suppression of cyclooxygenase-2 (COX-2) (60). The cyclooxygenases are involved in prostaglandin synthesis. Additionally, 5-lipoxygenase is a RORα1 target gene, presenting with a RORE site (55, 56). The lipoxygenase is an enzyme crucial to the formation of leukotrienes. Again, the prostaglandin F2 receptor negative regulator (*PTGFRN*) and prostaglandin E synthase (*PTGES*) were both identified as RORα target genes during a genome-wide study on the human neuronal cell line SH-SY5Y (81). Essentially, the cyclooxygenases and lipoxygenases are enzymes that synthesize prostaglandins and leukotrienes from nutritionally essential fatty acids, namely, linoleic and α-linolenic acid, which go on to synthesize arachidonate, the precursor to prostaglandins, thromboxanes, and leukotrienes.

### RORα: thyroid hormones

Another HPA-controlled axis of hormones is the thyroxine-releasing hormone (TRH)–thyroid-stimulating hormone (TSH)–T3/T4 pathway. The transcript expression of *RORα* in the thyroid gland is at 12.8 nTPM as per the Human Protein Atlas (46). Boukhtouche et al. (2010) studied the importance of tri-iodothyronine (T3) as a mediator of dendritic branching in the cerebellum, through their studies on the RORα-deficient staggerer mice (17). Only the RORα1 isoform is regulated by tri-iodothyronine (T3), in the cerebellum of Swiss mice when compared to the staggerer mice, which failed to express RORα (17).

The TRH-TSH-T3/T4 pathway of hormones is one of the most important hormonal pathway in the HPA, controlling metabolism of carbohydrates, proteins, and lipids, and T3 has long been documented to play a pivotal role in metabolism at the cellular level (85, 86). Regulation of the thyroid gland is under the influence of the pituitary that secretes the TSH and the hypothalamus, which releases the TRH. The thyroid hormone receptor (THRB) gene is a potential RORα target, as identified during a genome-wide study on a neuronal cell line (81).

## RORα: metabolism

With developmental defects being the hallmark of the staggerer mice, the most significant study was that of Roffler-Tarlov and Sidman (1978), wherein they noted reduced glutamic acid, a prerequisite in the uninterrupted synthesis of the building blocks of the RORα gene: the purines adenine and guanine, and pyrimidines thymine and cytosine (**Figure 1**). Glutamic acid is a metabolic by-product of carbohydrate, lipid, and protein metabolism, the glutamine→glutamate→α-ketoglutarate cycle serving as the constant source of metabolic fuel that governs normal embryogenesis, growth, and development. As expected, the staggerer mice revealed aberrant metabolic signaling in addition to the observations of cerebellar defects and a staggering gait by Sidman et al. (1, 51–53, 87–93). Lau et al. (2008) observed that the staggerer mice were leaner, with reduced fat pads; Trenkner and Hoffman (1986) noted that the staggerer mice presented with smaller thymus and spleen when compared to their wild-type counterparts, and Lau et al. (2004) directly implicated RORα to lipid metabolism (7, 9, 42). These macroscopic, microscopic, and metabolic observations in the staggerer mice had set the stage for researchers looking for answers to the metabolic pathways affected (3, 5–7, 18, 25, 51–53, 56–59, 70, 79, 84, 87, 94–98). The staggerer mice exhibit altered protein, lipid, and glucose metabolism (3, 5–7, 25, 42, 94). In support of the above statement, Kang et al. (2011) noted that the levels of serum cholesterol, HDL, and glucose were lower in the staggerer mice, with implications on a homeostatic mechanism to control adiposity/weight (5, 7, 9, 42, 56). Liver being one of the most metabolically active organs serves as a hub for control of lipid, protein, and glucose metabolism (95, 98). One of the liver enzymes *PNPLA3* (1-Acylglycerol-3-Phosphate O-Acyltransferase), a RORα target gene, is regulated through a c-JUN-RORα-PNPLA3 pathway and is inactivated in the presence of a high-fat diet (97). Several other phospholipase proteins, *PNPLA2, PNPLA7*, and *PNPLA8*, were identified as RORα target genes (81). Mice on a high-fat diet undergo hepatic stress, which leads to an activation of c-Jun and the subsequent inactivation or deregulation of RORα-PNPLA3 (97). Essentially, the cJun : RORα:PNPLA3 pathway is activated under hepatic duress or an overload of a diet rich in fats, and a study on the HepG2 cells identified significance for the RORα:HIF1α and RORα:VEGF pathways (54, 97). RORα4 is the predominant isoform in the liver of mice and has also been validated on the HepG2, human liver cells (99). *Cyp7b1* was identified as a RORα target in the liver and the cytochromes, a group of heme-like enzymes that are present abundantly in the liver (59). The liver, being a metabolically active organ, is a hub for the storage, distribution, and utilization of carbohydrates, lipids, and proteins, including disposal of metabolic waste. The cytochromes, which are also integral to the respiratory chain, are essential to carbohydrate, lipid, and protein metabolism. Carbohydrate metabolism-associated genes/proteins, PEPCKc, and Glucose-6-Phosphatase (G6PC) are dysregulated in the staggerer mice (95). Most of the dietary non-essential proteins give rise to intermediates of the Krebs tricarboxylic citric acid cycle, and the dietary intake of a “nutritionally balanced diet” is assimilated into the various organs and tissues through the metabolic enzymes involved in carbohydrate, protein, and lipid metabolism.

### Staggerer mice: lipid metabolism

Not long after the discovery of a mutant RORα being identified as the cause of developmental defects in the staggerer mice, Patrick Lau et al. (2004, 2008), through a series of extensive studies, published their research entitled “RORalpha regulates the expression of genes involved in lipid homeostasis in skeletal muscle cells (2004)”, after studying several genes involved in lipid metabolism within the mice myogenic cell line C2C12 (7, 42). The prelude to the studies by Patrick Lau et al. were the discoveries by Bertin et al. (1990), Vu-Dac et al. (1997), and Raspe et al. (2001) (5, 7, 42, 51, 53). Bertin et al. (1990), during studies on the staggerer mice, noted differential expression/levels of the BAT and their energetic metabolism at 28°C, when compared to nonmutant controls, the adipose tissue being the main storage tissue for lipids (5). Vu-Dac et al. (1997) and Raspé et al. (2001), while studying metabolism, identified RORα as a transcriptional regulator of apolipoprotein A-I (*APOA1*) and apolipoprotein C-III (*APOC3*), the transporters of lipids in blood (51, 53). Patrick Lau et al. (2008) subsequently published their work on the role of lipid metabolism in the staggerer mice and summarized their findings with the following sentence: “In summary, our study suggests RORα is an important factor in the regulation of genes associated with lipid homeostasis and adiposity” (7). The staggerer mice were leaner and were obesity resistant, the pads of WAT and BAT offering the staggerer mice a better chance of survival (5, 7). Moreover, the expression of *ADRB2*, an adrenergic receptor, was increased in the WAT, BAT, and skeletal muscle of staggerer mice (7). The levels of plasma triglycerides, non-esterified fatty acids (NEFA), total cholesterol, and high-density-lipoprotein (HDL) were reduced in the staggerer mice (7). Additionally, expression of RORα1 and RORα4 was also reduced in the liver, WAT (epididymal fat), and skeletal muscles of the staggerer mice (7). Expression profiling on genes involved in lipid metabolism and transport indicated differential transcript levels in the staggerer mice, with *SREBP1c* and *FAS* reduced in the liver, while there was an increase in *PGC1α* and *lipin 1* (7). The relative expressions in the WAT of the staggerer mice for *PGC1α* were higher, while the BAT expressed higher levels of *PGC1β*, although *PGC1α, PGC1β*, and *lipin1* were increased in the BAT and WAT (7). The skeletal muscles had reduced levels of *SREBP1c, GcK*, and *FAS* in the staggerer mice when compared with WT mice (7). The adipose tissue and skeletal muscles are also lipid storage tissues, during increased dietary intake of carbohydrates, proteins, and lipids. The staggerer mice also displayed lower levels of plasma triglycerides and apo C-III levels when compared with the WT (51). The decrease in plasma lipoproteins and apo C-III corresponds to lower mRNA levels of *apo C-III* in the liver and proximal and distal intestines of the staggerer mice (51). The lipids, cholesterol, and cholesteryl esters are transported in plasma as water-miscible lipoproteins, which are composed of the protein moiety termed apolipoprotein, and these apolipoproteins can function as ligands, enzyme cofactors, and enzyme inhibitors. The staggerer mice, when maintained on a high-fat atherogenic diet, elucidated reduced plasma levels of apolipoproteins, Apo-AI and Apo-AII (87). Further inspection identified reduced expression of the *Apo-A1* mRNA in the intestinal cells of the staggerer mice (53, 87). Indeed, Apo-A1 is a RORα1 target gene and contains a RORE site within the promoter region of the intestinal caco-2 cells studied (53). The RORα1 and RORα4 isoforms of RORα regulate the transcriptional activity of apolipoprotein A5 (*ApoA5*) in HepG2 and HUH7 cells; however, RORα has no effect on ApoA5 in the staggerer mice (52). A compensatory mechanism exists within the staggerer mice, whereby metabolic genes are dysregulated to overcome the loss of RORα activity. Additionally, it was revealed that there was an association between hypo-alphalipoproteinemia and decreased high-density lipoprotein (HDL) levels due to a downregulation of the *apoA-I* gene in the intestine only, with the *apoA-II* being unaffected at the genetic level in the intestine (87).

Delving further into the role of RORα in lipid metabolism, crucial enzymes involved in the cholesterol and fatty acid synthesis pathway, namely, acetyl-coenzyme A acetyltransferase 1 precursor (*ACAT1*), acetyl coenzyme A acetyltransferase 2 (*ACAT2*), and short-chain acyl-CoA dehydrogenase precursor (*ACADS*), were identified as potential RORα target genes in the Sarachana and Wu genome-wide study (81). Kang et al. (2011) also defined a prominent role for RORα in lipid metabolism (56). Studies on the RORα staggerer (RORA sg/sg) mice demonstrated that genes involved in lipid metabolism, like *GPAM/GPAT1, AGPAT9, CIDEC, CIDEA*, and *MOGAT1*, were significantly downregulated (7, 42). The genes are direct targets of RORα (64). In the liver of RORα staggerer mice, several other genes involved in fatty acid metabolism were suppressed, including *ACOT3* and *ACOT4* (acyl-coenzyme A thioesterases), *FABP5, ADFP/PERILIPIN 2, LPIN2, ANGPT14*, and *FGF21* (7, 42). After extensive studies on the staggerer mice, the levels of biochemical lipids in the serum of the staggerer mice, and specific sets of metabolic enzymes, Patrick Lau et al. (2008) wrote: “The orphan nuclear receptor, RORalpha, regulates gene expression that controls lipid metabolism”. Evidence for the above statement lies in the fact that α-ketoglutarate in the glutamine→glutamate→α-ketoglutarate cycle is central to the Krebs citric acid cycle, and acetyl-CoA along with oxaloacetate biosynthesize citrate, catalyzed by citrate synthase (**Figure 1**). Citrate synthase had been identified as a RORα target gene and the enzymatic activity was diminished in the staggerer mice (100). More importantly, the lipid metabolites that are crucial to the glutamine→glutamate→α-ketoglutarate cycle are acetyl-CoA, malonyl-CoA, propionyl-CoA, and HMG-CoA, with Kallen et al. (2002, 2004) having identified cholesterol, the Acetyl-CoA→HMG-CoA→squalene pathway metabolite, as a natural ligand of RORα (19, 20).

### Staggerer mice: carbohydrate metabolism

After delineating the role of RORα in lipid metabolism, Patrick Lau et al. (2011) delved into studying the role of RORα in carbohydrate metabolism, through an understanding of the role of insulin in glucose uptake by the tissues in the staggerer mice when compared to wild-type littermates (94). The staggerer mice exhibited increased insulin sensitivity upon being challenged with intraperitoneal insulin on time-course assays (94). Glucose tolerance tests on the staggerer versus wild-type control mice detected an enhanced ability of glucose clearance from the blood within 90 min in the staggerer mice (94). The skeletal muscles being a suitable tissue for studies on glucose metabolism, utilization, gluconeogenesis, glycogenesis, and glycogenolysis have been used as an ideal tissue to study the effect of mutations and hormones (94, 95). The glucose uptake transporter Glut4 was significantly increased in the skeletal muscles of staggerer mice studied, and the increase corresponded to an increase in Akt and that of phosphorylated Akt upon stimulation with insulin (94). A noteworthy observation was that the glucocorticoid-inducible kinase 1 (*Sgk1*) transcript was upregulated in the skeletal muscles of the staggerer mice, with Sgk1 being considered as a regulator of transport channels (95). A regulator of energy metabolism, *PGC1β* was differentially regulated in the skeletal muscles and liver of staggerer mice when compared to the wild-type littermates, with reduced expression being observed in the skeletal muscles (7). Basically, the skeletal muscles serve to store excess blood glucose as glycogen. While insulin enhances glucose uptake and glycogen synthesis by the skeletal muscles, glucagon and epinephrine (a metabolite of the essential amino acid phenylalanine) promote the breakdown of glycogen into readily available forms of glucose (101). Glucose-6-phosphatase (G6PC), an enzyme essential in the breakdown of glycogen→glucose-6-phosphate→glucose, is regulated by RORα (95). The staggerer mice had reduced expression of *G6PC* and *PCK1*, both of these being involved in gluconeogenesis/glycogenolysis (95). Apart from the staggerer mice being sensitive to insulin, the adrenergic receptor β2 (*ADRB2*) was also observed to be upregulated in the skeletal muscles of staggerer mice (7). Nevertheless, the importance of carbohydrate metabolism in the staggerer mice and embryogenesis lay in the biochemical evidence that pyruvate and α-ketoglutarate are the center points in the synthesis of glutamine: the crux in purine and pyrimidine synthesis (**Figure 1**).

### RORα: the mitochondria

The staggerer mice expressed differential amounts of BAT and WAT, which is an indicator of the number of mitochondria within these specific cell types (5). Yang et al. (2006) noted differential expression of several oscillatory genes within the BAT and WAT of mice (40). RORα was the predominant oscillatory gene in the WAT of control mice, with the relative mRNA levels fluctuating between the Zeitgeber hours of 0 to 24 (40). Upon mining for information using the UCSC genome browser, a study for the protein RORα yielded information regarding an interaction with nucleoside diphosphate kinase, nm23-2 (28). Nucleoside di-phosphate kinase enzyme (NDPK) isoforms are essential to cellular differentiation and proliferation, and thereby in organogenesis; while *nm23-M1, nm23-M2*, and *nm23-M3* were expressed in the cerebellum and cerebral cortex, *nm23-M4* is present in the proliferating layer during organogenesis (70). Basically, the NDPK are a group of enzymes that catalyze conversion of diphosphate energy molecules into their triphosphate form, e.g., UDP is catalyzed to UTP and ADP is catalyzed to ATP. Interestingly, genes involved with components of the ATP synthase and ATPase genes—ATP synthase H+ transporting, mitochondrial F0 complex subunit F (*ATP5J*), ATPase H+/K+ transporting subunit alpha (*ATP4A*), ATPase plasma membrane Ca2+ transporting 2 (*ATP2B2*), ATPase phospholipid transporting 11A (*ATP11A*), and probable cation-transporting ATPase 13A3 (*ATP13A3*)—were identified as putative RORα targets (81).

The mitochondrion, the powerhouse of a cell, serves as the source of adenosine triphosphate (ATP) synthesis, and the ATP synthase complex along with ATPase act as its mediators. More specifically, ATP, the energizer of biochemical reactions within cells, is synthesized by the ATP synthase complex composed of several subunits spanning the mitochondrial membrane (102). The genome-wide study on a neuronal cell line identified adenylate cyclase (*ADCY5*) and ADP-dependent glucokinase (*ADPGK*) as putative target genes of RORα (81). The oxidative phosphorylation in the respiratory chain, of which ATP is a component, requires oxygen, and hypoxic conditions lead to defective synthesis of ATP; the requirement of ATP is essential in the synthesis of lipids, carbohydrates, and proteins (103). Hypoxia induces several signaling pathways, some of which are the HIF, PI3K/AKT/mTOR, MAPK, and NFκB pathways (104, 105). It is the *RORα4* isoform that is activated under hypoxic conditions, and the effect is through molecular level interactions regulating the hypoxia-responsive element/site (106). Miki et al. (2004) had concluded that the RORα4 promoter is a site that is activated under hypoxic stress and its activation is through the HIF-1 and Sp1/Sp3 pathway (107). While Chauvet et al. showed that RORα is a HIF-1 target gene, Kim et al. demonstrated that melatonin-induced RORα activates HIF-1α in a dose-dependent manner (54, 108). The transcript levels of *RORα1* and *RORα4* increased progressively under hypoxic conditions (107, 108). A study on keratinocytes showed that under hypoxic conditions, gene silencing of RORα led to a reduction in the expression of *HIF1α*, the hypoxia-associated gene (108). In essence, RORα is a crucial factor in cellular stress response/hypoxia, and the expression of RORα1 and RORα4 increased proportionately to HIF1α and VEGF, upon activation with melatonin on a time-course assay (54, 106–108). Thus, it appears that there is a fine cross-talk of positive and negative feedback circuits that dictate the regulation of both RORα and hypoxia-associated genes.

### Staggerer mice: protein metabolism

Protein metabolism is at the core of all metabolic pathways, since their by-product acts as enzymes, and of the genes/proteins regulated. Glutamate, a metabolite of glutamic acid, which is also derived from essential amino acids histidine and arginine, was reduced in the cerebellar cortex and cerebellar deep nuclei of the staggerer mice (**Figure 1**) (3). Glutamate is encoded by GAA and GAG, a set of three purines. The cerebellar cortex in the staggerer mice was underdeveloped and postnatal development led to degenerated granule cells and dendritic branching was affected (17, 108). The building blocks of DNA, the purines and pyrimidines, are all synthesized through an enzymatic pathway that requires, as precursors, glutamine, glycine, and aspartate in addition to ATP, carbohydrate metabolic intermediates, folates, and vitamins. An aberrant purine metabolic pathway could also lead to transcriptional and translational errors, more so with respect to genes spanning several thousands of kilobase pairs.

The metabolic hormone tri-iodothyronine (T3), a proteinaceous hormone, is unable to induce dendritic differentiation in the staggerer mice when compared to the wild-type mice (17). The muscles and bones are assimilated due to a homeostatic protein metabolic machinery and has been discussed in the “Staggerer mice: bone and muscle development” section.

Embryogenesis and the development of an embryo into a foetus, is the culmination of an interaction between several molecular and metabolic processes, which finally organize themselves into the various organs. An embryo is composed of mainly proteins, and proteins are composed of amino acids that organize themselves as RNA/DNA; RNA/DNA is composed of the purines and pyrimidines; the staggerer mice had reduced glutamic acid and glutamine is not only essential to the synthesis of purines and pyrimidines, but also integral to carbohydrate, lipid, and protein metabolism through the glutamine→glutamate→α-ketoglutarate cycle (**Figure 1**) (3). The staggerer mice thus developed due to an endocrine–molecular circuitry being disrupted during embryogenesis (1, 2, 90–92, 109).

## RORα: HPA-staggerer mice—embryogenesis and development

An embryonic failure during the developmental stages of life led to the birth of the staggerer mice, displaying a staggering gait, a grossly underdeveloped cerebellar cortex, thin long bones, and smaller thymus and spleens (1, 2, 9). The underdeveloped cerebellum was noticeable at birth in the staggerer mice, which clearly indicated that RORα dictated crucial molecular–metabolic–HPA networks during embryogenesis (1, 2, 109). Notwithstanding the experimental evidence from studies on the levels of glutamic acid in the staggerer mice by Roffler-Tarlov and Sidman (1978), it is an embryonic level HPA-like circadian rhythm that regulates embryogenesis (3, 109). RORα acts as a “molecular–circadian regulator”; a deletion to RORα’s LBD leads to molecular events during embryogenesis that affect the development of the cerebellum, among few other organs (1–4, 9). A global gene expression profiling study on the developmental stages of a mouse embryo revealed that *RORα* was one of the genes enriched during the Theiler stages TS22, TS23, TS25, and TS27 (109). The mice embryonic stages during which RORα was differentially expressed corresponded to the human embryonic developmental stages of neurulation and organogenesis (109).

### Staggerer mice: neural development

Embryogenesis is a well-organized set of stages, with one set of activated genes, relaying the information onto another subset of crucial genes, the end result being a fetus without mutations. Any disruption to the relay of genomic information, via mutations, leads to clearly identifiable characteristics in the newborn. Likewise, RORα was crucial to the patterning of cells during dendritic branching in the staggerer mice (17, 110, 111). Landis and Sidman noted that all cerebellar granule cells, present at day 3, degenerate by day 28 of postnatal development in the staggerer mice (2). Indeed, a study of the cerebellar cortex, conducted on staggerer mice within the ages of 12 days to 3 months, revealed a delay in the development of defective dendritic cell spines (14, 17, 90). Moreover, the tyrosine intermediate tri-iodothyronine (T3) has been studied as an activator of RORα, committed to dendritic differentiation (17). Since the staggerer mice have evident deformities within the cerebellar cortex, RORα serves as a stem cell marker, responsible for the development of a well-organized cerebellum and cerebellar cortex (14, 17, 111). The expression of *RORα* reduces between embryonic stages E15.5, E17.5, and postnatal day 0, in the cerebellum of staggerer mice when compared to the wild type; this reduction is followed by reduced expression of Shh, Gli1, and Ptch at E15.5, E17.5, and P0 (44). While studying neurovascular coupling, Ye Sun et al. (2017) noted a reverse relationship between RORα and the semaphorins SEMA3a, SEMA3c, SEMA3d, and SEMA3e (61). While RORα was downregulated in the staggerer mice, the semaphorins (*SEMA3a, SEMA3c, SEMA3d*, and *SEMA3e*) were upregulated when compared to the wild type (61). The semaphorins play an important role in the central nervous system (112). The expression of SEMA7A increases exponentially from the embryonic stages (E15 and E19) to postnatal stages (P5 and P14) in rat brains (113). Microarray analysis of P5 and P6 staggerer versus wild-type mice brains demonstrated reduced levels of semaphorin *SEMA7A, ADCY8, NEPH*, and *NR2F1* in the cerebral cortex, along with a reduction in the expression of *Netrin G1, CD47, CSPG5*, and *NEPH* in the ventro-basal thalamic nuclei of the staggerer mice (111). Dendritic branching and cortical thickness were altered in the staggerer mice (110, 111). The study by David Gold et al. (2009) illustrated the sequential activation of groups of sets of genes from the embryonic E12.5 stage until postnatal day 4, with RORα beginning to express itself within the cerebellum in wild-type mice at E12.5 (44). The David Gold study (2009) on staggerer mice cerebella identified a clear association of aberrant RORα signaling, with effects on the PIM1, HIST2, and IDB2 (44). IDB2 (Inhibitor of DNA binding 2) is rhythmically expressed in the SCN of the cerebella (114). RORα is expressed in the SCN, a circadian rhythm pacemaker, and also acts as a molecular–circadian regulator during embryogenesis (14, 17, 67). A circadian whole genome expression analysis on mice identified the ataxin genes *Atxn1, Atxn2, Atxn3, Atxn7*, and *Atxn10* as oscillatory genes (47). A characteristic of the Atxn deficiency is that it leads to ataxic movements, as had been observed in the staggerer mice (1, 2, 49, 115, 116). A mutation to any of the crucial circadian genes and their regulatory pathways would lead to ataxic movements. A study by Sarachana and Wu (2013) identified *ATXN7L3* and *ATXN7L1*, two of the ataxin 7-like genes, as RORα targets in a neuronal cell line (81).

### Staggerer mice: bone and muscle development

Ataxic movements and the stagger were a characteristic of the staggerer mice (1, 2). Two years since the discovery of a mutant RORα being the reason for the staggerer mice, Thomas Meyer et al. (2000) published his work implicating RORα in bone metabolism (6). Bones in the staggerer mice were long, thin osteopenic bones, with reduced bone mineral content and lowered bone density (6). Additionally, experiments focused on studying the role of RORα in bone metabolism delineated its involvement in osteogenic differentiation. More specifically, there was enhanced expression of RORα in the mesenchymal stem cells of the bone marrow (6). Enhanced expression of RORα1 was observed in the human MG-63 osteoblasts (117). Min et al. (2019) defined the role for E2-mediated activation of RORα in osteoblast differentiation (18). An overexpression of RORα1 led to subsequent increases in the expression of bone markers, alkaline phosphatase (ALP), osteocalcin (OC), and collagen type I (117). RORα is activated during osteogenic differentiation, in addition to RORα regulating the promoters of the mouse sialoprotein (BSP) (6). A cholesterol metabolite, 1α, 25-dihydroxyvitamin D3 (Calcitriol), a known regulator in bone pathology, was identified as a ligand of RORα (20).

With an aim to elucidate the functional role of RORα1 in the skeletal muscle, Lau et al. (1999) demonstrated the significance of myogenin and p21, in myogenesis and differentiation (50). *RORα1* and *RORα4* are both downregulated in the quadriceps skeletal muscle of staggerer mice, along with *SREBP1c* and *PGC1β* being downregulated (7). Myogenin (*MyoG*), Troponin 1 (*TNNI1*), and troponin 2 (*TNNI2*) followed *RORα*’s expression trend, during differentiation of proliferating myoblasts into post-mitotic differentiated myotubes (42, 50). It was the PI3K-AKT pathway that was the predominantly upregulated pathway, in the skeletal muscles of the staggerer mice (94). A separate study noted that the myogenic C2C12 cells express RORα with the RORα1 isoform interacting noncompetitively with p300 and MyoD (50). Additionally, a microarray analysis of human skeletal muscles stimulated with testosterone demonstrated the differential regulation of *RORα* (82). Thus, RORα is a multifunctional protein, with its most distinguished role during embryogenesis, which had led to the birth of the staggerer mice (1–4).

### Staggerer mice: HPA and immunity

The bones, in particular the bone marrow, thymus, spleen, and kidneys are considered as the hematopoietic organs, and the hematopoietic cells released into the vasculature act as the sentinels that offer immunity. Macroscopic studies on the staggerer mice revealed smaller thymus and spleens, while the lymph nodes were swollen, when compared to WT littermates (9). Trenkner and Hoffmann (1986) write about the similarities between the cerebellar and thymic development, in that both these organs develop around the 10th day of embryonal organogenesis (9). Antibodies were produced by the spleen cells of the staggerer mice, although the antibody titers decline with a longer lag phase than do their WT littermates (9). There is a fine network between cerebellar functions, HPA, circadian rhythm, and immunity that was highlighted between the 1970s and the 1990s, when studying the effects of sleep deprivation (118–122). The effects of sleep on circulating human lymphocyte population in a circadian manner have been studied extensively (120, 123, 124). Stoyan Dimitrov et al. (2009) illustrated their observations on the circadian rhythm of T lymphocyte subsets very clearly in the form of a graphical representation for each of the CD4+ and CD8+ T cell subsets: naïve, effector, and memory (124). The human lymphocytes could be considered as cellular glands, due to the several cytokines secreted by each subset of cell. The soluble mediators cytokines, chemokines, and thrombopoietins have been researched to activate circadian transcription factors, with IL-33, CCL7, and IL-6 known to activate RORα (125, 126). The naïve CD4+ and CD8+ T cells peak during the early hours between 2 and 5 a.m., followed by a trough at around 11 a.m (124). The circadian pattern is similar for the naïve, effector, and memory CD4+ and CD8+ T cells, although the sigmoidal pattern is most pronounced for the naïve CD4+ and CD8+ T cells (124). The cortisol, epinephrine, and norepinephrine concentrations follow the reverse trend, with a dip between 2 and 5 a.m. and a peak between 8 and 11 a.m (124). Constant perturbances to the sleep–wake cycles lead to an increased susceptibility to infections. In a study by Palmblad et al. in 1979, researchers demonstrated in 12 young male volunteers that depriving them of sleep resulted in reduced cell-mediated immunity (118). Prior to this, Akerstedt and Torsvall noted that personnel working night shifts had a predisposition to taking more sickness leaves (127). A heightened immune response to the Sendai virus was observed when involved in stressful vigils (118). On a study involving six male volunteers, altered immune functions along with increased levels of IL-1 were observed during sleep cycles (128). A subsequent study on 40 h of a wake period revealed aberrant immune responses with an increase in IL-1-like and IL-2-like activity (129). The review by Harvey Moldofsky details the studies regarding suppressed immune response observed in sleep-deprived animals and humans performed between 1969 and 1993 (130). Furthermore, a comprehensive study by Joyce et al. detailed the role of working conditions on the health of personnel (131). Referring back to the above studies, and the significance of these observations to RORα, during a study on 12-h light and dark cycles, differential expression of RORα and the melatonin receptor (MTNR3) was observed in the thymocytes, lymph node CD4+ T cells, splenic B200+ B cells, and the bone marrow of mice (132).

With a clear cascade of associations between the circadian rhythm, sleep, melatonin, and immunity, studies on the RORα-deficient staggerer mice gained momentum. A study of dark room lighting versus brighter rooms for better health of personnel working in the nursing division defined a role for the circadian hormone melatonin (133, 134). Melatonin exerts its functions as an immunomodulator through its receptors, presented on cells that elicit a melatonin-inducible response (133). Carrillo Vico et al. (2004) demonstrated through *in vitro* assays that both the naïve and activated human lymphocytes synthesize and secrete melatonin, far above those obtained from physiologic levels in the human serum (71). The human lymphocytes are cellular endocrine/exocrine glands, which also produce melatonin (16, 71, 73, 135, 136). Furthermore, there was a correlation between the release of melatonin and IL-2: an inhibition of melatonin synthesis led to a decrease in the production of IL-2, and *vice versa* (71). Melatonin, the circadian regulated hormone, has been shown to enhance the production of IL-2, IFNγ, and IL-6 in humans and activate RORα (15, 16, 134, 135). Interestingly, seasonal variations in immunity were observed in mice injected with melatonin (137).

RORα presents with beneficial effects in an immunocompetent individual, as opposed to those who are immunocompromised; however, the staggerer mice presented with exceptional immuno-modulatory mechanisms that protected them from endotoxic shock (138). The staggerer mice were resistant to endotoxic shock due to reduced IL-6, IL-1β, CCL2, and TNFα, upon stimulation with lipopolysaccharide (LPS) (138). Specific immune cell populations respond differentially in the staggerer mice. The neutrophil numbers are enhanced in bronchoalveolar fluids from LPS-induced lungs of staggerer mice (139). Interestingly, the staggerer mice when challenged with ovalbumin (OVA) presented with reduced eosinophils and neutrophil numbers within the bronchoalveloar fluid (140). Moreover, the concentration of cytokines IL-4, IL-5, IL-13, and eotaxin 1 (CCL11) was reduced in the OVA-stimulated staggerer mice (140). The macrophages in staggerer mice produce enhanced amounts of IL-1 upon being challenged with LPS and are sensitive to external stimuli (141, 142). Additional studies on the staggerer mice noted reduced expression of the IL-2 receptor (*CD25*) and *SCA1* in the lungs of staggerer mice when compared to the WT (143). Again, it was the large intestine of staggerer mice, an organ that harbors microbial flora, where the expression of *CD25* and *Sca1* was reduced, followed by a concomitant enhanced expression of *CD127* and *c-kit* (143). The addition of IL-25 to small intestine biopsies failed to trigger an IL-5 increase in the staggerer mice (143). Addition of IL-25 in the mesenteric lymph nodes of the staggerer mice failed to enhance the number of nuocytes or CD4+ T cells, although nuocytes in the wild-type mice increased by seven- to eightfold (144). RORα is expressed abundantly in the nuocytes and is required for nuocyte development (144). The staggerer mice presented with severely reduced nuocyte numbers in the bone marrow, while the T cells were barely affected (144). RORα is also expressed in the Th2 and Treg cells, as demonstrated by single-cell RNA-sequencing (scRNA-seq) on CD4+ T cells in mice infected with *Nippostrongylus brasiliensis* (125). The increase in RORα corresponds to increases in IL1rl1 and CXCR6 (125). A separate study detected an upregulation followed by a downregulation of RORα upon progression of CD8+ T cells from naïve, to effector, memory, progenitor dysfunctional, early dysfunctional, and late/terminal dysfunctional stages (145). The *Clock* gene presented a similar expression profile to *RORα* in the CD8+ T cells (145). RORα is highly expressed in the KLRG1^hi^ CD8+ effector T cells when compared to the KLRG1^lo^ CD8+ effector T cells (146). The Th17 lineage of T cells presented with upregulated *RORα* expression even in the OT-II mice upon stimulation with splenic antigen presenting cells and OVA peptide (147). Additionally, RORα was upregulated by TGFβ and IL-6 in activated CD4+CD25-CD62L^hi^ CD44^lo^ T cells from mice (147). However, RORα1 upon activation by TNFα promotes repression of IL-6 and IL-8 (60). Naïve CD4+ T cells from the staggerer mice exhibited reduced expression of the IL-23 receptor and IL-17, along with an increase in the expression of the transcription factor T-bet, when stimulated with TGFβ, IL-6, TNFα, IL-1β, IL-23, αIL-4, and αIFNγ (147). The study defined a role for the IL-6:STAT3 pathway in the regulation of RORα within CD4+ T cells (147). Haim-Vilmovsky et al. (2021) comprehensively studied the expression of *RORα* in various T-cell subsets (125). I had previously identified a TTCNNNGAA (GAS sequence) within the first intron of RORα as a STAT5a target site using the ChIP cloning–sequencing strategy in human CD8+ T lymphocytes during my doctoral studies between 2006 and 2009 (148).

## RORα and the staggerer mice: some other facts

RORα is expressed not only in the skeletal muscles but also in the smooth muscles of the heart and intestines, as depicted by Delerive et al. (2001) (60). More specifically, RORα is expressed in the smooth muscle cells from saphenous veins, coronary artery smooth muscle cells, and the human aortic smooth muscle cells (60). The staggerer mice present with reduced expression of smooth muscle (SM)-myosin, calponin, and heavy (h)-caldesmon in the mesenteric arteries (149). The melatonin-RORα pathway protects the heart, which is composed of smooth muscles, from myocardial ischemia/reperfusion injury (8). RORα also protects cardiomyocytes against angiotensin II-mediated myocardial hypertrophy (150). Moreover, RORα also protects the kidneys against renal ischemia/reperfusion injury (151). It was the smooth muscle hub—the small intestines of the staggerer mice—that exhibited RORα-mediated expression of *apoA-1* (53). *RORα* acts as a multi-functional protein with differential activity in various tissues.

## RORα: details of sequence and loci identified as a Stat5 target site in CD8+ T cells [from my doctoral research and thesis]

**Figure 5:**
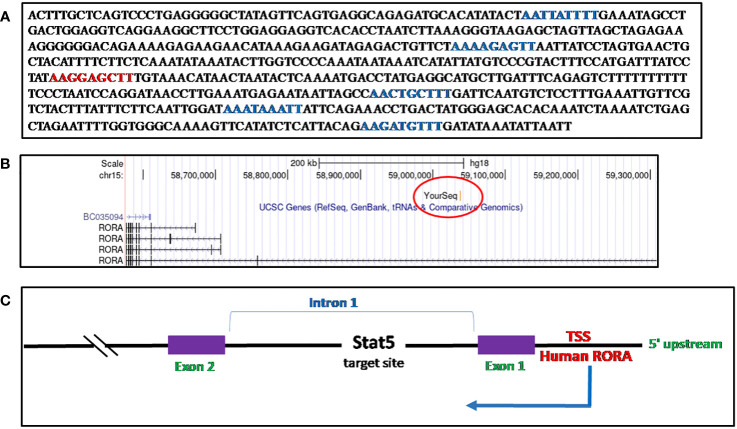
Sequence of RORα in activated CD8+ T cells and mapping. **(A)** The Stat5 target sequence identified through ChIP cloning and sequencing. There is one consensus GAS motif (TTCNNNGAA) site, denoted in red, while there are five other half-GAS sites (TTNNNNNAA), denoted in green. **(B)** The RORα sequence identified as a STAT5 target gene in human CD8+ activated T cells through a ChIP-cloning strategy maps within the first intron, on the UCSC Genome Browser, Human Mar. 2006 (NCBI36/hg18) build (28). **(C)** A schematic representation of the Stat5 target sequence within the RORα gene.

I identified a STAT5a target sequence in phytohemagglutinin (PHA)-activated human CD8+ T cells, through ChIP cloning and sequencing, which mapped to the first intron of *RORα1* (148). The sequence, when blasted onto the UCSC Genome Browser, March 2006 (NCBI36/hg18) build, identified the loci as Chr15:59037073-59037676 (Chr15q22.2), *RORα*1 (28, 147) (**Figures 5A–C**, **Supplementary Figure 1A**). There are five half GAS sites (TTNNNNNAA) and one TTCNNNGAA (GAS) site within the 599-base pair sequence (28, 150) (**Figure 5A**). It was during a set of time-course experiments on IL-2 stimulated preactivated CD8+ and CD4+ T cells, performed to study the expression of the STAT5a/STAT5b target genes identified, where *RORα* was detected as unique from the other genes, in that, upon stimulation with IL-2, RORα downregulated itself sequentially by more than twofold during a 6-hour time course (148). A separate study on STAT5 target genes in human CD4+ T cells identified *RORα* to be a candidate gene (152). Previous studies had validated the regulation of RORα through the STAT3 pathway (126, 153).

## Discussion and conclusion

I identified a sequence within intron 1 of the *RORα* gene as a STAT5a target site in human PHA-activated CD8+ T lymphocytes; its unique expression profile on a time-course experiment in interleukin-2 (IL-2) stimulated activated CD8+ T cells, and publications on the dysfunctional RORα staggerer mice are the basis for the contents in this review (1–7, 9, 13, 14, 83, 87, 89–94, 138, 141, 148, 149).

RORα could be denoted as a molecular–circadian regulator due to its predominant role during embryogenesis. It was a deletion of 160 kilobases, encompassing the *RORα* gene that led to the development of the staggerer mice. A genome-wide analysis of the staggerer mice at the time of its discovery (Sidman et al, 1962) could have helped understand the complex association between defects observed within the cerebellar cortex, the gait (stagger) observed, and the other known/unknown morphological/pathological/biochemical changes associated with the genetic insertions/translocations/deletions identified (1). The stagger and the underdeveloped cerebellar cortex during the developmental stages in the staggerer mice were obvious indicators that suggested a role for RORα in embryogenesis. Subsequent studies on the effects of a dysfunctional RORα in metabolism, osteogenesis, and skeletal and smooth muscles, including immunity, laid the foundation that indicated RORα was a multi-functional protein, with its main function materializing during the embryonic stages of life (1–9, 13, 14, 42, 50, 83, 87, 89–94). More precisely, Boukhtouche et al. (2006) and Vitalis (2018) mapped the differentiation of Purkinje cells and barrel cortex development, with neurogenesis being one of the first organs to be organized during embryogenesis (110, 111). Sato et al. in 2004 very aptly suggested the existence of RORα as a core component of the circadian rhythm (21). Thus, RORα stands at the crossroads, being regulated by the ligands and regulating genes involved in embryonic patterning during organogenesis. Apart from hormones as the ligands of RORα, other soluble mediators like cytokines produced by hematopoietic cells also serve as ligands of RORα (16–18, 125, 126). Although the main ligands of RORα are active metabolites like melatonin, T3, and cholesterol intermediates, it appears that RORα functions as an enzyme, similar to Fisher’s substrate-enzyme model wherein cholesterol serves as a substrate to competitively inhibit the binding of other ligands (substrates like melatonin, T3, E2, and corticosterone), with cholesterol having been discovered to be occupying the LBD of RORα (16–20, 97). It could be reasoned that the proteinaceous hormones activate RORα, while cholesterol occupies the LBD and maintains RORα in a resting phase (inactive), akin to the G0 cell-cycle phase.

Several transcription factors and ligands control the functions of RORα, sometimes exhibiting redundant and, at other times, unique roles. The redundancy of RORα’s functions to other transcription factors, like REV-ERBα and REV-ERBβ, is specific only to their common redundant roles in specific organs and tissues (10–12). For example, a compensatory mechanism exists within the staggerer mice; since these mice already presented with a stagger, they were prevented from diet-induced obesity, through a compensatory-dysregulated metabolic profile in the staggerer mice (1–7, 9, 13, 14, 83, 87, 89–94, 139, 142, 149). These altered metabolic effects may have been endowed onto the staggerer mice as a homeostatic mechanism to be able to cope with the defective locomotive function. Again, the uniqueness of RORα’s regulatory activity stems from its differential expression in specific organs; for example, the cerebellum in the brain presents with the highest expression of RORα (46).

Interestingly, Roffler-Tarlov and Sidman (1978) observed lowered glutamate levels in the staggerer mice, glutamate being a precursor in the synthesis of purines and pyrimidines and thus DNA/RNA. Since all the amino acids, which are the building blocks of proteins, are composed of a triplet code, composed of three of the five purines and pyrimidines, adenine (A), guanine (G), thymine (T), cytosine (C), and uracil (U), a defective organization of the nucleotide sequence and/or a deficiency of an essential amino acid could result in mutations. Most of the mutant mice with distinct macroscopic features have been identified with mutations within genes spanning more than 350,000 base pairs (28, 49). Examples of these mice are as follows: (1) The lurcher mice, with a GRID2 mutation within a gene spanning 1,412,259 base pairs, were born with an evident wobbly lurching gait. (2) The scrambler mice also suffer from an ataxic gait and the causation has been defined as a mutation within the DAB1 gene spanning 377,255 base pairs. (3) The reeler mice with a mutation within the RELN gene, spanning 460,249 base pairs (28, 49). A defective cell cycle and DNA replicative machinery, due to scarcity of dietary essential amino acids, an unbalanced diet, or a defective metabolic enzymatic machinery, will possibly lead to deletions of large fragments of DNA and thus mutations. However, with these observations by Roffler-Tarlov and Sidman (1978) on the staggerer mice, none of the subsequent studies ever addressed the importance of nutritionally essential fatty and amino acids, carbohydrates, and lipids, and how a titrated addition of these even after birth could affect the postnatal development of the staggerer mice (1). **Figure 1** highlights the importance of the observations by Roffler-Tarlov and Sidman (1978) (3).

Of interest is also the fact that mice are nocturnal and although RORα was also identified as a circadian gene, RORα may have identical roles in humans and mice, in a diurnal–nocturnal manner. While RORα in humans is transcribed in a 5′–3′ direction towards the centromere, in the mouse, it is transcribed in a 5′–3′ direction towards the telomeric end. Thus, it could be probable that RORα may be regulated in humans and mice in an identical manner, albeit the expression of RORα in humans during the day is similar to the expression of RORα in mice during the night and *vice versa* (28).

**Figure 6:**
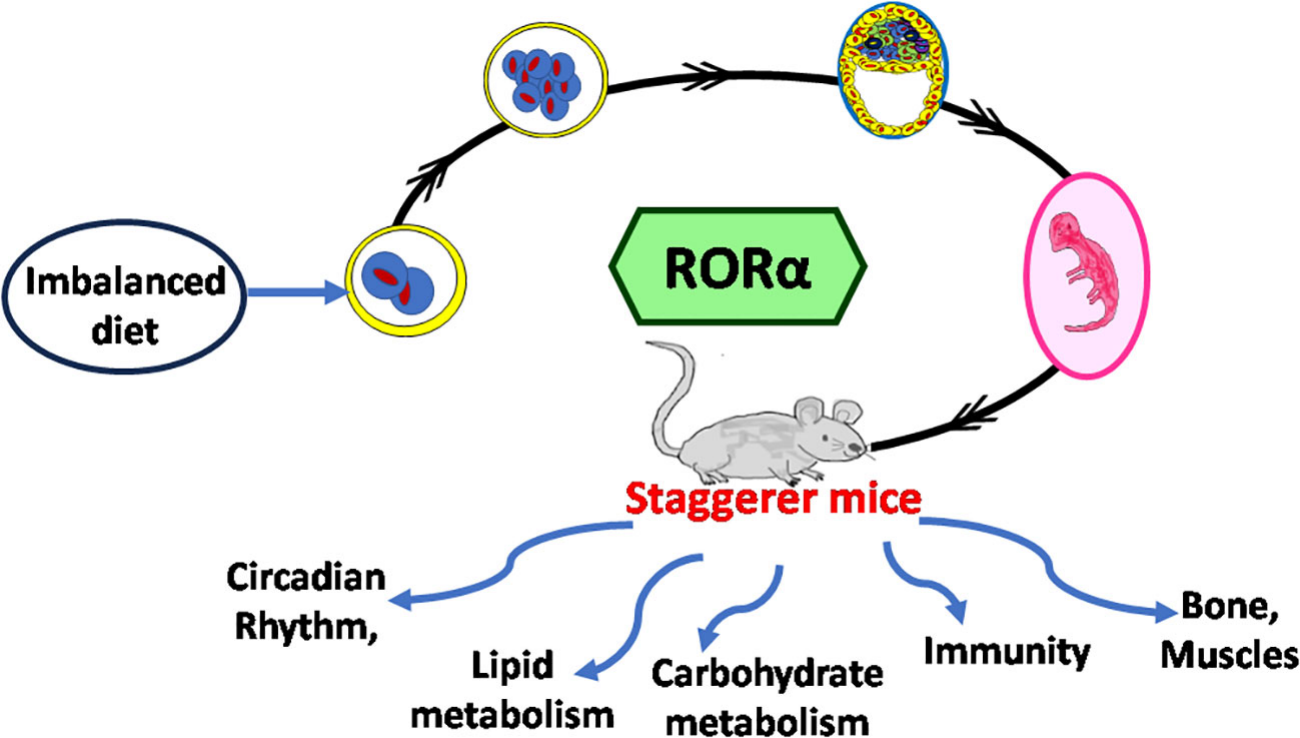
Conclusion and discussion, behind the discovery of the staggerer mice. Nutrition is the hallmark of an effective transcriptional system, uninterrupted by mutations accumulated due to the absence of essential amino acids and a balanced diet. This then promotes embryogenesis devoid of mutations. In the absence of essential nutrients in a diet, the stages of embryogenesis are affected, thereby leading to mice with a stagger. Researchers used the staggerer mice to study circadian rhythmicity, lipid metabolism, carbohydrate metabolism, bone mineralization, immunity and the compensatory mechanisms within muscles, bone mineralization, circadian rhythmicity, metabolism, and immunity. At the center of all the research studies was a deletion encompassing the ligand binding domain of RORα.

It was during my doctoral studies on the “Identification and Analysis of IL-2 induced STAT5 target genes in Human CD4 and CD8 T cells” that I noted a decline in the expression of RORα on a time-course experiment, unlike the other genes identified as STAT5 target genes. I have since then noted RORα as a protein distinct from the other proteins and thus this review. A summary of the story behind the birth of the staggerer mice is depicted in **Figure 6**.

## Important notes

With the staggerer mice exhibiting a smaller and leaner body mass than the wild-type mice, and studies having substantiated an altered metabolic profile when compared to the wild-type control mice, a study of the various hormones that could possibly regulate RORα would be interesting.

Subsequent studies, on a supposedly “staggerer mice”, must be whole genome sequenced to identify its similarity to the first staggerer mice identified in 1962, with the 160-kb deletion affecting the LBD of *RORα*.

## Author contributions

AR: Conceptualization, Writing – original draft, Writing – review & editing.

## Supplementary material

Supplementary Table 1 Nuclear receptor groups [Collated and reproduced from HUGO gene nomenclature committee].

Supplementary Table 2 Some genes regulated by RORα

Supplementary Figure 1 STAT5 target site. **(A)** Mapping the sequence onto the UCSC Genome browser [Human Mar. 2006 (NCBI36/hg18) identified the loci of the sequence on the chromosome 15 (28). **(B)** Custom track view of conservation within mammals of the TTN5AA and TTCNNNGAA sequences (28).
