# Supplementary material for: RAR-related orphan receptor alpha and the staggerer mice: a fine molecular story
Source: Front Endocrinol (Lausanne). 2024 May 3;14:1300729. doi: 10.3389/fendo.2023.1300729 (PMC11099308; doi:10.3389/fendo.2023.1300729)
Supplement: Supplementary Table 1 — Nuclear receptor groups [Collated and reproduced from HUGO gene nomenclature committee]. [file DataSheet_1.docx]

**Supplementary table 1: Nuclear receptor groups [Collated and reproduced from HUGO gene nomenclature committee].**

| **Nuclear Receptors** | **Nuclear receptor subgroup** | **Nuclear receptor subfamily** | **Chromosome** |
| --- | --- | --- | --- |
| Group 1 |  | Nuclear receptor subfamily 0 group B member 1 (NR0B1/DAX/ AHCH) | ChrX: p21.2 |
|  |  | Nuclear receptor subfamily 0 group B member 2 (NR0B2/SHP) | chr1: p36.11 |
| Group 2 | Thyroid hormone receptors | thyroid hormone receptor alpha (THRA/ EAR-7.1/EAR-7.2/THRA3/AR7/ERBA/ NR1A1) | Chr17: q21.1 |
|  |  | thyroid hormone receptor beta (THRB/ THRB1, THRB2, NR1A2, THR1, ERBA-BETA, GRTH) | Chr3: p24.2 |
|  | Retinoic acid receptors | retinoic acid receptor alpha (RARA/ RAR/NR1B1) | Chr17:q21.1 |
|  |  | retinoic acid receptor beta (RARB/ HAP/NR1B2/RRB2) | Chr3: p24.2 |
|  |  | retinoic acid receptor gamma (RARG/ RARC/NR1B3) | Chr12:q13.13 |
|  | Peroxisome proliferator activated receptors | peroxisome proliferator activated receptor alpha (PPARA, hPPAR, NR1C1) | Chr 22:q13.31 |
|  |  | peroxisome proliferator activated receptor delta (PPARD/ NUC1/NUCII/FAAR/NR1C2 | Chr 6: p21.31 |
|  |  | peroxisome proliferator activated receptor gamma (PPARG/PPARG1/PPARG2/NR1C3/ PPARgamma | Chr 3: p25.2-p25.1 |
|  | RevERBalpha/RevERBbeta | Nuclear receptor subfamily 1 group D member 1 (NR1D1/ ear-1/hRev/Rev-ErbAalpha/THRA1/REVERBA/REVERBalpha) | Chr17:q21.1 |
|  |  | nuclear receptor subfamily 1 group D member 2 (NR1D2/ BD73/RVR/EAR1r/HZF2/Hs.37288/ REVERBB/ REVERBbeta | Chr3: p24.2 |
|  | Liver X receptors | liver X receptor-alpha /nuclear receptor subfamily 1 group H member 3 (NR1H3/ LXR-a/RLD-1/LXRa) | Chr 11: p11.2 |
|  |  | liver X receptor-beta/ nuclear receptor subfamily 1 group H member 2 (NR1H2/ NER/NER-I/RIP15/LXR-b/LXRb) | Chr 19: q13.33 |
|  |  | farnesoid X receptor - nuclear receptor subfamily 1 group H member 4(NR1H4/ FXR/RIP14/HRR1/HRR-1) | Chr 12:q23.1 |
|  |  | nuclear receptor subfamily 1 group H member 5, pseudogene (NR1H5P/ Fxrb/NR1H5) | Chr 1:p13.2 |
|  | RAR related orphan receptors | RAR related orphan receptor A (RZRA/ROR1/ROR2/ ROR3/NR1F1/RORΑ) | Chr15:q22.2 |
|  |  | RAR related orphan receptor B (RORB/ RZRB/ NR1F2/ ROR-BETA) | Chr 9:q21.13 |
|  |  | RAR related orphan receptor C (RORC/ RZRG/RORG/ NR1F3/ TOR) | Chr 1:q21.3 |
|  | Retinoic acid receptors | Vitamin D receptor (VDR/ NR1I1/ PPP1R163) | Chr12:q13.11 |
|  |  | pregnane X receptor (NR1I2/ ONR1/PXR/BXR/SXR/PAR2) | Chr 3:q13.33 |
|  |  | nuclear receptor subfamily 1 group I member 3 (NR1I3/ MB67/CAR1/CAR) | Chr 1:q23.3 |
| Group 3 | Hepatocyte nuclear factor 4 receptors | hepatocyte nuclear factor 4 alpha (NR2A1/HNF4/HNF4A/TCF14/MODY/ MODY1) | Chr20: q13.12 |
|  |  | hepatocyte nuclear factor 4 gamma (NR2A2/HNF4G) | Chr 8:q21.11 |
|  | Retinoid X receptors | retinoid X receptor alpha (RXRA, NR2B1) | Chr9:q34.2 |
|  |  | retinoid X receptor beta (RXRB/ NR2B2/H-2RIIBP/RCoR-1) | Chr6: p21.32 |
|  |  | retinoid X receptor gamma (RXRG/ NR2B3) | Chr 1: q23.3 |
|  | NR2C family | Nuclear receptor subfamily 2 group C member 1 (NR2C1/TR2-11) | Chr 12: q22 |
|  |  | nuclear receptor subfamily 2 group C member 2 (NR2C2/TAK1/TR2R1/hTAK1) | Chr 3: p24.3 |
|  | NR2E family | Nuclear receptor subfamily 2 group E member 1 (NR2E1/TLL/XTLL) | Chr6: q21 |
|  |  | nuclear receptor subfamily 2 group E member 3 (NR2E3/PNR/rd7/RP37) | Chr15:q23 |
|  | NR2F family | Nuclear receptor subfamily 2 group F member 1 (NR1F1/EAR-3/COUP-TFI/TCFCOUP1/ SVP44/COUPTF1) | Chr 5:q15 |
|  |  | nuclear receptor subfamily 2 group F member 2 (NR2F2/COUP-TFII/ COUPTFB/SVP40/NF-E3/COUPTF2) | Chr15: q26.2 |
|  |  | nuclear receptor subfamily 2 group F member 6 (NR2F6/EAR-2/EAR2) | Chr 19: p13.11 |
| Group 4 | Estrogen receptors | estrogen receptor 1 (ESR1/ NR3A1/Era/ER-alpha) | Chr 6: q25.1 |
|  |  | estrogen receptor 2 (ESR2/ NR3A2/Erb/ ER-beta | Chr14: q23.2 |
|  | Estrogen related receptors | estrogen related receptor alpha (ESRRA/ ERR1/ERRalpha/ NR3B1/ ERRa) | Chr 11:q13.1 |
|  |  | estrogen related receptor beta (ESRRB/ ERR2/ ERRbeta/NR3B2/ ERRb) | Chr 14:q24.3 |
|  |  | estrogen related receptor beta gamma (ESRRG/ NR3B3/ERRg/ ERR-gamma) | Chr 1:q41 |
|  | Glucocorticoid receptor (NR3C1/GR) |  | Chr 5:q31.3 |
|  |  | Nuclear receptor subfamily 3 group C member 2 (MR, NR3C2) | Chr 4:q31.23 |
|  | Progesterone receptor (PGR/ NR3C3) |  | Chr 11:q22.1 |
|  | Androgen receptor (AR/AIS/NR3C4/SMAX1/HUMARA) |  | Chr X:q21 |
| Group 5 |  | Nerve growth factor IB/ nuclear receptor subfamily 4 group A member 1 (NGFIB/ NR4A1/TR3/N10/NAK-1/NGFIB/NUR77) | Chr 12: q13.13 |
|  | Nuclear receptor related 1 protein (NURR1/ NR4A2/ TINUR/NOT/RNR1/HZF-3) |  | Chr 2:q24.1 |
|  | Neuron-derived orphan receptor 1 (CSMF/CHN//NOR1/MINOR) |  | Chr9:q31.1 |
| Group 6 | Steroidogenic factor-1 (SF-1, NR5A1, FTZ1, ELP, AD4BP, hSF-1) |  | Chr9:q33.3 |
|  | Liver receptor homolog-1 (NR5A2/ FTZ-F1beta/hB1F/LRH-1/FTZ-F1/hB1F-2/B1F2/LRH1) |  | Chr1:q32.1 |
| Group 7 | GC nuclear factor (GCNF/GCNF1/NR6A1/ RTR/CT150) |  | Chr 9:q33.3 |

**Supplementary Table 2: Some genes regulated by RORα**

|  | **Treated with/ Stages studied** | **Model in which studied (Cells/Staggerer mice** | **Type of assay** | **Ref** |
| --- | --- | --- | --- | --- |
| CAV-3 |  | COS-1 , C2C12 cells | Luciferase assay, RT-PCR | 45 |
| CIDEC |  | staggerer mice | qRT-PCR | 57 |
| CIDEA |  | staggerer mice | qRT-PCR | 57 |
| GPAM/GPAT1 |  | staggerer mice | qRT-PCR | 57 |
| AGPAT9 |  | staggerer mice | qRT-PCR | 57 |
| MOGAT1 |  | staggerer mice | qRT-PCR | 57 |
| ACOT3 |  | staggerer mice | qRT-PCR | 57 |
| ACOT4 |  | staggerer mice | qRT-PCR | 57 |
| FABP5 |  | staggerer mice | qRT-PCR | 57 |
| ADFP/PERILIPIN 2, |  | staggerer mice | qRT-PCR | 57 |
| LPIN2, |  | staggerer mice | qRT-PCR | 57 |
| ANGPT14 |  | staggerer mice | qRT-PCR | 57 |
| FAS |  | C2C12 cells | qRT-PCR | 45 |
| ADRP |  | C2C12 cells | qRT-PCR | 45 |
| ACS4 |  | C2C12 cells | qRT-PCR | 45 |
| SCD1 |  | C2C12 cells | qRT-PCR | 45 |
| SCD2 |  | C2C12 cells | qRT-PCR | 45 |
| SREBP1 |  | C2C12 cells | qRT-PCR | 45 |
| VEGF | melatonin | HEP G2 cells | Luciferase, western blotting, siRNA | 55 |
| HIF1α | melatonin | HEP G2 cells | Luciferase, western blotting, siRNA | 55 |
| ALOX5 |  |  | electromobility shift assay | 56 |
| ApoAI |  | staggerer mice, Caco2 | Gel retardation, | 54 |
| APOA5 |  | HepG2, HUH7 | Gel retardation, qRT-PCR | 31 |
| AMPK | Cholesterol sulfate |  | western blotting, Luciferase assay | 59 |
| LXRα | Cholesterol sulfate |  | western blotting, Luciferase assay | 59 |
| APOCIII |  | HepG2, Caco2, RK13,  staggerer mice | Northern blotting, Luciferase assay | 28 |
| SHH | developmental stages | staggerer mice | ChIP | 47 |
| SLC1a6 | developmental stages | staggerer mice | ChIP, RT-PCR | 47 |
| ITPR1 | developmental stages | Cerebellum, staggerer mice | ChIP, RT-PCR | 47 |
| PCP4 | developmental stages | Cerebellum, staggerer mice | ChIP | 47 |
| PCP2 | developmental stages | Cerebellum,  staggerer mice | ChIP | 47 |
| ACOT3 |  | staggerer mice | qRT-PCR | 57 |
| ACOT4 |  | staggerer mice | qRT-PCR | 57 |
| FABP5 |  | staggerer mice | qRT-PCR | 57 |
| LPIN2 |  | staggerer mice | qRT-PCR | 57 |
| ANGPT14 |  | staggerer mice | qRT-PCR | 57 |
| FGF21 |  | HEK293 cells, staggerer mice | qRT-PCR, luciferase assay | 57 |
| CYP19A1 |  | THP-1, HUVEC | ChIP assay, Luciferase assay | 58 |
| MIF |  | THP-1, HUVEC | ChIP assay, Luciferase assay | 58 |
| ABCA1 |  | THP-1, HUVEC | ChIP assay, Luciferase assay | 58 |
| Sema3a |  | staggerer mice | qRT-PCR | 90 |
| Sema3c |  | staggerer mice | qRT-PCR | 90 |
| Sema3d |  | staggerer mice | ChIP assay, qRT-PCR | 90 |
| Sema3e |  | staggerer mice | ChIP assay, qRT-PCR | 90 |
| SEMA7A |  | staggerer mice | Microarray | 89 |
| NEPH |  | staggerer mice | Microarray | 89 |
| ADCY8 |  | staggerer mice | Microarray | 89 |
| NR2F1 |  | staggerer Mice | Microarray | 89 |
| Netrin G1 |  | staggerer Mice | Microarray | 89 |
| CD47 |  | staggerer Mice | Microarray | 89 |
| CSPG5 |  | staggerer Mice | Microarray | 89 |
| CYP7B1 |  | staggerer Mice | qRT-PCR, luciferase assay | 60 |
| IkBα |  | PAC1A | Luciferase assay, EMSA | 61 |
| PNPLA3 | High fat diet | Mice hepatocytes | Western blotting, qRT-PCR | 113 |

**Supplementary Figure 1**

A.


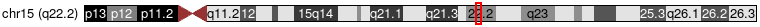


**B**


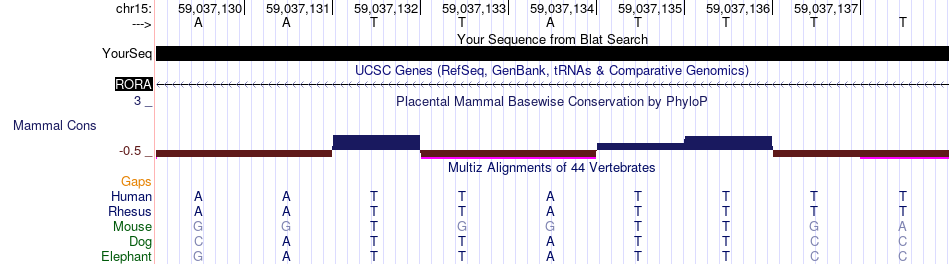

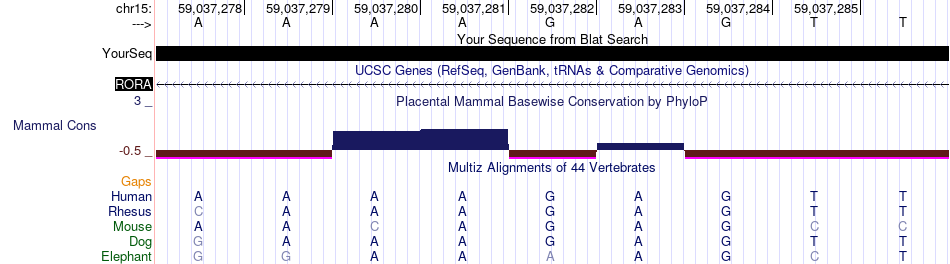

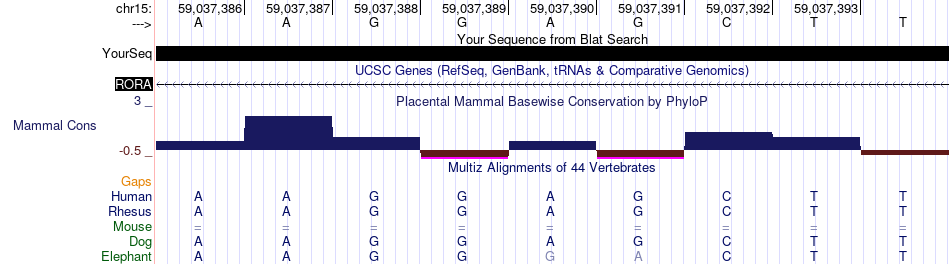

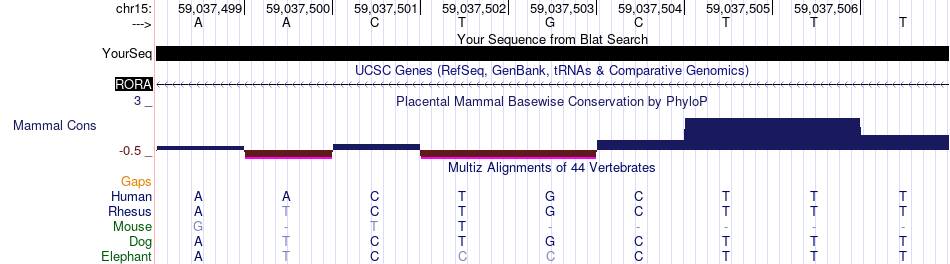

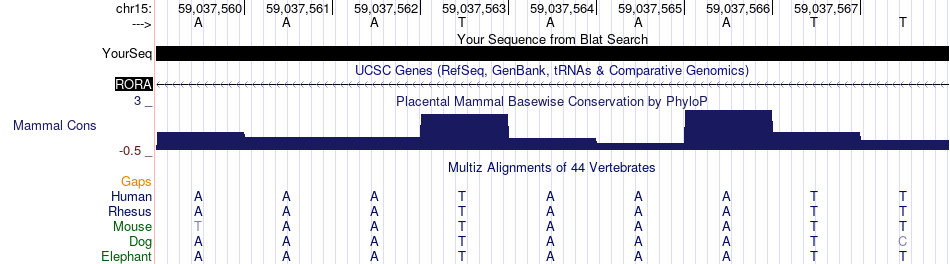

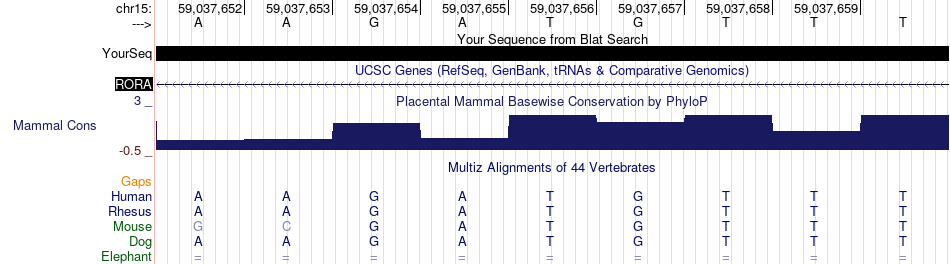


chr15:59,037,652-59,037,660

chr15:59,037,560-59,037,568

chr15:59,037,499-59,037,507

chr15:59,037,386-59,037,394

chr15:59,037,278-59,037,286

chr15:59,037,130-59,037,138

**Supplementary figure 1: STAT5 target site.** **A.** Mapping the sequence onto the UCSC Genome browser [Human Mar. 2006 (NCBI36/hg18) identified the loci of the sequence on the chromosome 15 [27]. **B.** Custom track view of conservation within mammals of the TTN5AA and TTCNNNGAA sequences [27].
